# Knowledge guides attention to goal-relevant information in older adults

**DOI:** 10.1186/s41235-021-00321-1

**Published:** 2021-08-18

**Authors:** Maverick E. Smith, Lester C. Loschky, Heather R. Bailey

**Affiliations:** Department of Psychological Sciences, Kansas State University, 471 Bluemont Hall, 1100 Mid-campus Dr., Manhattan, KS 66506 USA

**Keywords:** Aging, SPECT, Eye movements, Event comprehension, Attention

## Abstract

How does viewers’ knowledge guide their attention while they watch everyday events, how does it affect their memory, and does it change with age? Older adults have diminished episodic memory for everyday events, but intact semantic knowledge. Indeed, research suggests that older adults may rely on their semantic memory to offset impairments in episodic memory, and when relevant knowledge is lacking, older adults’ memory can suffer. Yet, the mechanism by which prior knowledge guides attentional selection when watching dynamic activity is unclear. To address this, we studied the influence of knowledge on attention and memory for everyday events in young and older adults by tracking their eyes while they watched videos. The videos depicted activities that older adults perform more frequently than young adults (balancing a checkbook, planting flowers) or activities that young adults perform more frequently than older adults (installing a printer, setting up a video game). Participants completed free recall, recognition, and order memory tests after each video. We found age-related memory deficits when older adults had little knowledge of the activities, but memory did not differ between age groups when older adults had relevant knowledge and experience with the activities. Critically, results showed that knowledge influenced where viewers fixated when watching the videos. Older adults fixated less goal-relevant information compared to young adults when watching young adult activities, but they fixated goal-relevant information similarly to young adults, when watching more older adult activities. Finally, results showed that fixating goal-relevant information predicted free recall of the everyday activities for both age groups. Thus, older adults may use relevant knowledge to more effectively infer the goals of actors, which guides their attention to goal-relevant actions, thus improving their episodic memory for everyday activities.

**Supplementary Information:**

The online version contains supplementary material available at 10.1186/s41235-021-00321-1.

## Significance statement

Although older adults tend to perform worse than young adults on tests of their episodic memory, research suggests that older adults may use intact knowledge to offset impairments in episodic memory. Further, knowledge of an activity affects where people attend. Can older adults use their experience to offset age-related declines in attention and everyday memory when viewing familiar everyday activities? We showed both young and older adults videos of actors performing activities, such as balancing a checkbook and setting up a video game console, while we tracked their eye movements. We asked whether people would pay more attention to goal-relevant actions (e.g., entering a balance into a checkbook) when they had more knowledge of the activity than when the activity was less familiar. We found that older adults looked less at important goal-related information (e.g., connecting the game console to the Wi-Fi network) when they had less knowledge of the activity, but they showed similar patterns of attention to young adults when they had more knowledge of the activity (e.g., balancing a checkbook). Furthermore, those young and older adults who attended to goal-relevant information better recalled the activity. Our results have implications for future memory interventions. Specifically, in addition to training older adults to improve their declining cognitive abilities (e.g., working memory and attentional focus), interventions could also be developed to help older adults make connections between relevant existing knowledge and new information. Such an intervention should help direct attention to important, goal-relevant information, which could subsequently improve episodic memory for everyday activities.

## Introduction

Theory and intuition suggest that the more people know about a topic, the easier it is for them to encode, and later remember, information that accesses this knowledge. For instance, imagine watching a video, from a third-person perspective, of someone else preparing a cup of coffee or tea in the morning before going to work. Many people perform such an activity as part of their daily routine. You can easily understand and accurately remember details from the experience of watching someone else perform the activity if you can draw upon your prior knowledge for how to perform the necessary operations to complete the goals. By relying upon your prior knowledge and experience, you know what information is critical, and where to selectively attend to extract the important information needed to understand the task. You would be less likely to attend to the important information if you had no *schema* for the appliances and utensils needed to make hot beverages, or no *scripts* for how to make coffee or tea. The purpose of the current experiment was to evaluate the role of event knowledge on where people *look* (i.e., what they attend to), and whether this role changes with age. Thus, we will first describe a theoretical framework that posits an interplay between top-down and bottom-up processing on attention. Then, we will describe research evaluating top-down processing on attention in film. Finally, we will describe how these processes may be altered with age and how knowledge may affect these processes.

### The scene perception and event comprehension theory

The Scene Perception and Event Comprehension Theory (SPECT) is an integrative framework proposed, among other things, to explain how attention influences comprehension of visual events, and how comprehension, in turn, influences attentional selection (Loschky et al., 2018, 2020). SPECT distinguishes between back-end and front-end processes. Back-end processes are those involved in memory, and in particular, those that are involved in creating, maintaining, and storing *event models*—one’s online understanding of what is happening now. Event models are informed from both prior knowledge and the sensory input. Thus, one’s familiarity with an action should influence the contents of the event model when viewing a new instance of a familiar action. Front-end processes occur within single eye fixations—moments when the eyes are still. They include, among other processes, attentional selection. Attentional selection can be influenced by both *bottom-up* stimulus saliency (i.e., feature contrast in motion, brightness, orientation, size, etc.) and *top-down* cognitive processes such as the viewer’s event model (Henderson, 2007; Hutson et al., 2018).

### Bottom-up effects on eye movements

Studies have shown that computational models of visual saliency can predict where viewers look in scenes and videos (Itti & Borji, 2015; Itti & Koch, 2001). According to these models, attentional guidance is characterized predominantly as a reaction to the visual features of the stimulus, which pull attention to semantically uninterpreted image features (Henderson, 2007). However, higher-level features, such as the meaning of objects, also correlate substantially with visual salience (see Elazary & Itti, 2008; Henderson & Hayes, 2017; Tatler et al., 2011).

According to SPECT, moment-to-moment changes in where and when to move the eyes are directly linked to the viewer’s current event model (Loschky et al., 2020). Contrary to claims generated from SPECT, context manipulations, which show strong differences in viewers’ understanding of a film, fail to influence where and for how long viewers look. This effect has been called the *Tyranny of Film*, because viewers fixate many of the same places at the same time when watching a Hollywood film, despite having large differences in comprehension and memory of the clip (Davis et al., 2021; Hutson et al., 2017; Loschky et al., 2015; Smith & Mital, 2013). For instance, Hutson et al., (2017) manipulated viewers’ comprehension of a film by showing one group of participants a video of someone placing a bomb into a car trunk, before a couple, who did not know about the bomb, got into the car and drove away. The other group of participants did not see the beginning of the clip when the bomb was placed into the car. While the two groups had radically different event models of the film clip, as reflected by their predictions for what would happen next (i.e., the car will explode), their eye movements surprisingly showed strong attentional synchrony. People generally looked at the same places at the same time consistent with the Tyranny of Film. It was only when participants were given a volitional task of drawing a map of the scene that Hutson et al. (2017) observed differences in eye movements compared to when participants freely watched the clip. A variety of different studies have now found support for this lack of a mandatory top-down effect on eye movements in film (fandom, Huff et al., 2017; opinions about abortion, Hutson, 2018; tennis expertise, Taya et al., 2013). Further, Davis et al. (2021) found no age-related differences in gaze patterns between older and young adults despite significant differences in memory for a film (Davis et al., 2021). As such, bottom-up mechanisms, driven by motion signals, may play a dominant role in controlling where people attend, regardless of one’s understanding of a video (Hutson et al., 2017; Loschky et al., 2015; Mital et al., 2010).

### Top–down effects on eye movements

Eye movements can also be driven by higher-level cognitive processes, such as the meaning of a scene (i.e., the distribution of semantic information in a scene) (Hayes & Henderson, 2019; Henderson & Hayes, 2018; Henderson et al., 2009), observer’s tasks (Hutson et al., 2017; Yarbus, 1967), viewer perspective taking (Bacha-Trams et al., 2020; Lahnakoski et al., 2014), goals of individuals when they are performing actions (Tatler et al., 2013), and their event models (Hutson et al., 2018). Accordingly, attention is guided by the cognitive system to parts of the activity that are semantically informative and relevant to the observer (Henderson, 2007). Many studies in the text comprehension literature have demonstrated strong effects of the event model on eye movements during reading (see Rayner, 1998 for a review). For instance, readers make longer fixations, shorter saccades, and more regressive eye movements (eye movements to previously read text) when text becomes difficult to comprehend (Rayner, 1998, 2009), such as when the number or gender of a pronoun mismatches that of its referent (Ehrlich & Rayner, 1983), or when a protagonists’ action violates a predictive inference informed from the protagonists’ goal (Calvo et al., 2001).

Top-down effects, such as one’s understanding of a scene or comprehension of a text, affects eye movements when people view static images or read text, but there is less evidence to suggest that comprehension affects eye movements when people watch films (see however Eisenberg et al., 2018; Flanagan & Johansson, 2003; Hayhoe et al., 2012; Rotman et al., 2006; Tatler et al., 2013). Both text and static scenes often lack certain bottom-up features that are present in film, which may allow for the event model to guide attention. For example, films have motion, which captures attention (Abrams & Christ, 2003; Mital et al., 2010), but static scenes and texts do not. In addition, highly produced films, like the ones used in previous studies that found evidence of the Tyranny of Film (Davis et al., 2021; Hutson et al., 2017; Loschky et al., 2015), may contain film-specific features such as cuts, close-ups, and foregrounding of objects in the center of the screen, that exert strong bottom-up control on eye movements (Dorr et al., 2010; Loschky et al., 2015; Smith, 2012; Tatler, 2007). Thus, the Tyranny of Film may be due to unique characteristics present in Hollywood films.

Aside from motion, these film specific features are not found when people perform, or watch others perform, events in the real-world from a third-person perspective. To simulate real world experiences in the current experiment, we used short films of naturalistic activities that excluded many of the standard film editing techniques present in Hollywood style films. Thus, we hypothesized that the videos in the current study may afford a stronger influence of top-down processing on attention than what has been used in previous work (Hutson et al., 2017; Loschky et al., 2015; Huff et al., 2017; Davis et al., 2021). Further, evidence of the Tyranny of Film has exclusively come from studies with young participants (see however, Davis et al., 2021). It is possible that the event model may be more influential on older adults’ eye movements due to age-related declines in perception, attention, and memory as we will outline in the next section.

### Age-related changes in cognition

Many perceptual and cognitive abilities decline with age. For instance, older adults tend to perform more poorly on tests of inhibition, executive functioning, reasoning, processing speed, attention, and working and episodic memory, compared to young adults (Balota et al., 2000; Craik & Byrd, 1982; Foster et al., 1995; Park et al., 2002; Salthouse, 1992, 2000). Older adults also exhibit deficits in remembering details of specific episodes, such as words or pictures from a list, specific information from narratives, and even autobiographical memories (Levine et al., 2002). In addition, older adults are less able to selectively attend to relevant information and inhibit irrelevant information while performing tasks ranging from antisaccade tasks (Olincy et al., 1997) to comprehension tasks (Hasher & Zacks, 1988). These deficits are, in part, due to decline in top-down attentional control mechanisms (Borges et al., 2020; Madden & Whiting, 2004).

Despite such decline, semantic knowledge tends to be spared with age (Park et al., 2002). For example, older adults retain the ability to recall the steps associated with everyday actions (i.e., script knowledge) (Light & Anderson, 1983) and the ability to use schemas when reading texts (Arbuckle et al., 1990; Miller et al., 2004, 2006). They have a robust vocabulary (Bahrick & Hall, 1991), and typically show little or even no deficit in processing information at the situation model level during discourse comprehension (Radvansky, 1999; Radvansky & Dijkstra, 2007; Radvansky et al., 2001). Older adults may use intact knowledge to either eliminate age-related differences in encoding and memory performance altogether, or perhaps to even outperform young adults (see Umanath & Marsh, 2014 for a review). In addition, sometimes this reliance on prior knowledge can be very beneficial to older adults; however, at other times, it can have negative effects.

Prior knowledge may impair older adults’ memory and attention at times because of their inability to suppress contextual information when it is irrelevant to the task (e.g., Lustig & Jantz, 2015). Older adults are more likely to falsely recall words they did not encounter on a list of related words (the DRM paradigm) (Norman & Schacter, 1997). In addition, search for a target object in a scene given a preview is slower in older adults than young adults when the preview is inconsistent with the scene containing the target object (e.g., priming search for a breadbasket in a kitchen with a picture of a kitchen vs. a parking lot) (Borges et al., 2020). They are also more likely to produce confabulations of modified versions of fairy-tale stories that are consistent with their prior knowledge (Barba et al., 2010).

However, in many situations, prior knowledge facilitates cognitive processing in older adults. For instance, older adults tend to fill in the gaps when they fail to retrieve episodic information. They use prior knowledge to activate stereotypes to aid comprehension when they read texts or view videos (Mather et al., 1999; Radvansky et al., 2010). They also use prior knowledge when recalling modified versions of well-known fairy tales such that they are more likely to recall information consistent with the original well learned version (Barba et al., 2010). Further, they are also better able to remember information, such as grocery prices, when they are consistent with their prior knowledge. For instance, Castel (2005) had young and older adults study pictures of common grocery items, and items were either priced at market value or an unusual price. Young adults displayed better recall of unrealistic prices than older adults, but there was no age difference for realistic prices. Castel found that young adults benefitted from the use of knowledge as well, but to a lesser degree than older adults. Thus, older adults may rely on prior knowledge to compensate for decline in episodic memory (see Umanath & Marsh, 2014).

Although research demonstrates that knowledge improves memory performance (e.g., Bransford & Johnson, 1972; Chase & Simon, 1973; Connors et al., 2011; Ericsson & Kintsch, 1995), less is known about how knowledge affects moment-to-moment encoding processes, particularly in older adults. It is possible that knowledge enhances memory performance, in part, by facilitating how effectively information is encoded and represented (Anderson & Pichert, 1978; Chase & Simon, 1973; Miller & Stine-Morrow, 1998; Miller et al., 2006; Reingold et al., 2001a, 2001b). For instance, Smith et al. (2020) presented participants with self-paced slideshows that depicted complex activities that were more or less familiar to older and young adults. They recorded how long participants spent looking at each image and then tested the participants’ memory. Presumably, viewers slow down when there are important changes in an activity (e.g., when goals are completed) to update their event model of the situation (Haberlandt & Graesser, 1985; Hard et al., 2011; Kosie & Baldwin, 2019; Zwaan et al., 1995). Smith et al. (2020) found that both young and older participants spent longer time viewing images that contained important changes, but only when they had relevant knowledge of the activity depicted in the slideshow. Thus, having a richer event model of an on-going event influences *how long* viewers attend to critical information in a slideshow. Further, older adults recalled the information in the more familiar slideshows more accurately. Together with the similar viewing time results described above, this suggests that benefits in memory were due, in part, to changes that occurred in how people encoded the slideshows. Here, we extend the prior work by investigating *what information* viewers preferentially attend to when comprehending a new instance of activities they regularly perform.

### Goal structures and event comprehension

According to SPECT and other prominent models of event understanding (Gernsbacher, 1990; Kintsch, 1998; Zacks et al., 2007; Zwaan & Radvansky, 1998), comprehension of a naturalistic activity depends upon one’s ability to generate an event model of what is happening in the current event. Event models reflect various event dimensions, such as the spatial–temporal framework in which the actions take place, the agents involved in performing the action, and critically, the goals of the primary agents (Zwaan & Radvansky, 1998). In fact, many researchers have argued that event understanding revolves around keeping track of the goals and the plans of the agents involved (Suh & Trabasso, 1993; Trabasso & Nickels, 1992; Trabasso & Wiley, 2005). To understand a video of someone performing an action, one first needs to infer the goals of the actor (Flanagan & Johansson, 2003; Rotman et al., 2006); though, people tend to fixate more task irrelevant information when watching someone else perform an action than when they perform the action themselves (Tatler et al., 2013). Further, information related to the current goal is kept in a heightened state of activation while readers comprehend narratives (Radvansky & Curiel, 1998), possibly because the goal is kept active in working memory. Information related to the current goal is more accessible than information related to completed goals (Radvansky & Curiel, 1998; Suh & Trabasso, 1993). Further, when readers encounter changes in goals, processing demands and the likelihood that the change will be perceived as an event boundary increases (Kurby & Zacks, 2019; Zwaan & Radvansky, 1998; Zwaan et al., 1995).

A great deal is known about where people attend when they watch others perform actions to complete goals. Observers look at similar locations as the actor performing the action, and they make predictive eye movements to the locations where items will be moved before the item arrives at its destination (Eisenberg et al., 2018; Flanagan & Johansson, 2003; Hayhoe et al., 2012). They also make predictive eye movements to regions that are goal-relevant but are slower to do so when the actor’s goal is less predictable (Rotman et al., 2006). When observing a familiar action, the activity may be fairly predictable, in part, because observed actions are mapped onto one’s own motor representations for those actions (De Vignemont & Haggard, 2008; Wolpert & Flanagan, 2001); however, people presumably lack the appropriate motor representations when they have less knowledge of the actions. Thus, people may be more likely to track the goals of actors performing familiar than unfamiliar tasks when viewed from a third-person perspective. We explored this possibility in the current study.

Importantly, prior work has demonstrated that older adults may be less likely to track goal-relevant information compared to young adults. For instance, their predictions about the time course of observed actions are less precise than are young adults’ predictions (Diersch et al., 2012), which is consistent with prior work demonstrating that older adults may have difficulty maintaining and updating goal representations in their event models (Braver & West, 2008; Mayr, 2001). Conversely, there is also evidence that older adults have a preserved ability to process goal-relevant information (Magliano et al., 2012; Radvansky, 1999; Radvansky & Dijkstra, 2007; Radvansky et al., 1996). Typically, readers slow down when they encounter situational shifts in a text, such as a change in a protagonists’ goals. Older adults show similar slowdowns at situational shifts to young adults, which suggests that they are similarly sensitive to event-based changes in goals (Radvansky et al., 2001).

### Hypotheses

The above work suggests that goal structures are critical to event comprehension, but it is unclear whether older adults have difficulty perceiving that structure. We hypothesized that there would be age-related differences in the extent to which viewers track goal-relevant information. It is possible that young adults attend more to goal-relevant information than older adults, who do not encode the goal structure of an activity as efficiently (Diersch et al., 2012; Kurby & Zacks, 2019). Alternatively, young and older adults may not differ in their attention to goal-relevant information because (1) older adults have a preserved ability to process information at the event level (e.g., Radvansky, 1999) or (2) the dynamic nature of the stimuli produce strong attentional synchrony for both young and older adults (Davis et al., 2021; Hutson et al., 2017; Mital et al., 2010).

SPECT proposes that comprehension of a film influences where people attend (Loschky et al., 2020). Thus, we predicted that when people watch videos of everyday events, those with a richer understanding of the event, due to their knowledge, prior experiences, and stored motor representations (Wolpert & Flanagan, 2001) of the activity, would be more likely to track goal-relevant information in the event (as measured by eye movements). Further, we expect that knowledge-related effects on eye movements will be especially strong amongst older adults given that knowledge influences how they encode everyday activities (Smith et al., 2020). Alternatively, it is possible that we would find no effects of knowledge on eye movements given the dynamic nature of the stimuli, consistent with the Tyranny of Film (Hutson et al., 2017).

In addition, we know from prior work that knowledge improves memory performance (Chase & Simon, 1973) and that older adults may rely on knowledge to a greater degree than young adults when recalling information (Hess & Slaughter, 1990; Miller & Stine-Morrow, 1998; Miller et al., 2004, 2006). Thus, we predicted that memory performance in older adults would be equal to or greater than performance observed in young adults when older adults can use prior knowledge. Further, we predicted we would observe age-related differences in memory performance when older adults lacked prior knowledge of the activity (like effects reported in Arbuckle et al., 1990; Castel, 2005; Smith et al., 2020). Lastly, given that understanding of goals is critical to successful comprehension (Zwaan & Radvansky, 1998), we also predicted that attention to goal-relevant information would positively predict event memory.

## Method

### Participants

Sixty-three participants (*N* = 33 young adults, *N* = 30 older adults) participated in the experiment. No prior studies have examined the age-related effects of prior schemata on attention using naturalistic videos, but prior work has evaluated the effects of schemas on memory. Therefore, we conducted a power analysis in G*Power 3.1.9.6 using the estimate of effect size from Bransford and Johnson (1972). With an effect size of *d* = 0.77, alpha = 0.05, and power of 0.80, a total sample of 56 is needed. Thus, our study, with a sample size of 63 participants, should be adequately powered to detect the effects of interest on memory performance.

Young adults (16 females and 17 males) were recruited from Kansas State University’s Department of Psychological Sciences research pool and were compensated with course credit. Older adults (16 females and 14 males) were independent-dwelling people recruited from the local community. They were paid $10 per hour for their participation. Older adults completed a thorough phone screening prior to coming into the lab. They completed the AD8 screening interview (Galvin et al., 2005) and the Blessed dementia scale (Katzman et al., 1983) over the phone to screen for signs of dementia, and they provided information about their physical, mental, and neurological health. Exclusion criteria included several conditions that are associated with memory changes (e.g., neurological disorders, certain medications, concussions, etc.) or conditions that could affect the quality of the eye-tracking data (e.g., glaucoma, cataracts). Those who passed the screening measures were invited to participate in the experiment in the lab. In addition, both older and young adults completed the Mini Mental State Exam (Folstein et al., 1983) when they arrived in the lab. All the participants had scores above the minimal requirement (> 24). The experiment lasted approximately 2 h.

Participants completed a variety of individual difference measures throughout the study. Demographic information and performance on each of these measures are reported in Table 1. Young adults performed better in measures of processing speed, working memory, and event segmentation ability in the young adult activities, and older adults had significantly more years of education and vocabulary knowledge in line with other cognitive aging studies (e.g., Park et al., 2002).

**Table 1:** Means and standard errors for individual difference measures as a function of age

Measure (construct)	Young adult		Older adult		*t* value	*p*
	*M*	SE	*M*	SE		
Age	19.09	0.91	69.55	0.30	− 55.48	< .0001
MMSE	29.12	0.36	29.16	0.20	− 0.09	. 93
Years of education	13.17	0.21	16.69	0.45	− 7.15	< .001
*General semantic knowledge*						
Script generation	4.38	0.34	3.62	0.34	1.59	.12
Vocabulary	13.36	0.62	18.00	1.02	− 3.87	< .001***
*Processing speed*						
Pattern comparison	21.39	0.61	15.67	0.53	7.04	< .001***
Letter comparison	16.73	0.40	13.10	0.31	7.16	< .001***
DSST	39.64	1.64	32.63	1.41	3.24	< .01**
*Working memory*						
Rotation span absolute score	11.27	0.94	6.73	0.87	3.54	< .01**
Rotation span partial score	17.70	0.74	11.20	1.14	4.79	< .001***
*Segmentation agreement*						
Young adult activities	0.21	0.02	0.12	0.02	2.89	.02*
Older adult activities	0.21	0.02	0.17	0.02	1.38	.17

*p < .05; **p < .01; ***p < .001; MMSE = Mini Mental State Exam; DSST = Digit Symbol Substitution Task

### Materials

#### Videos

**Fig. 1:**
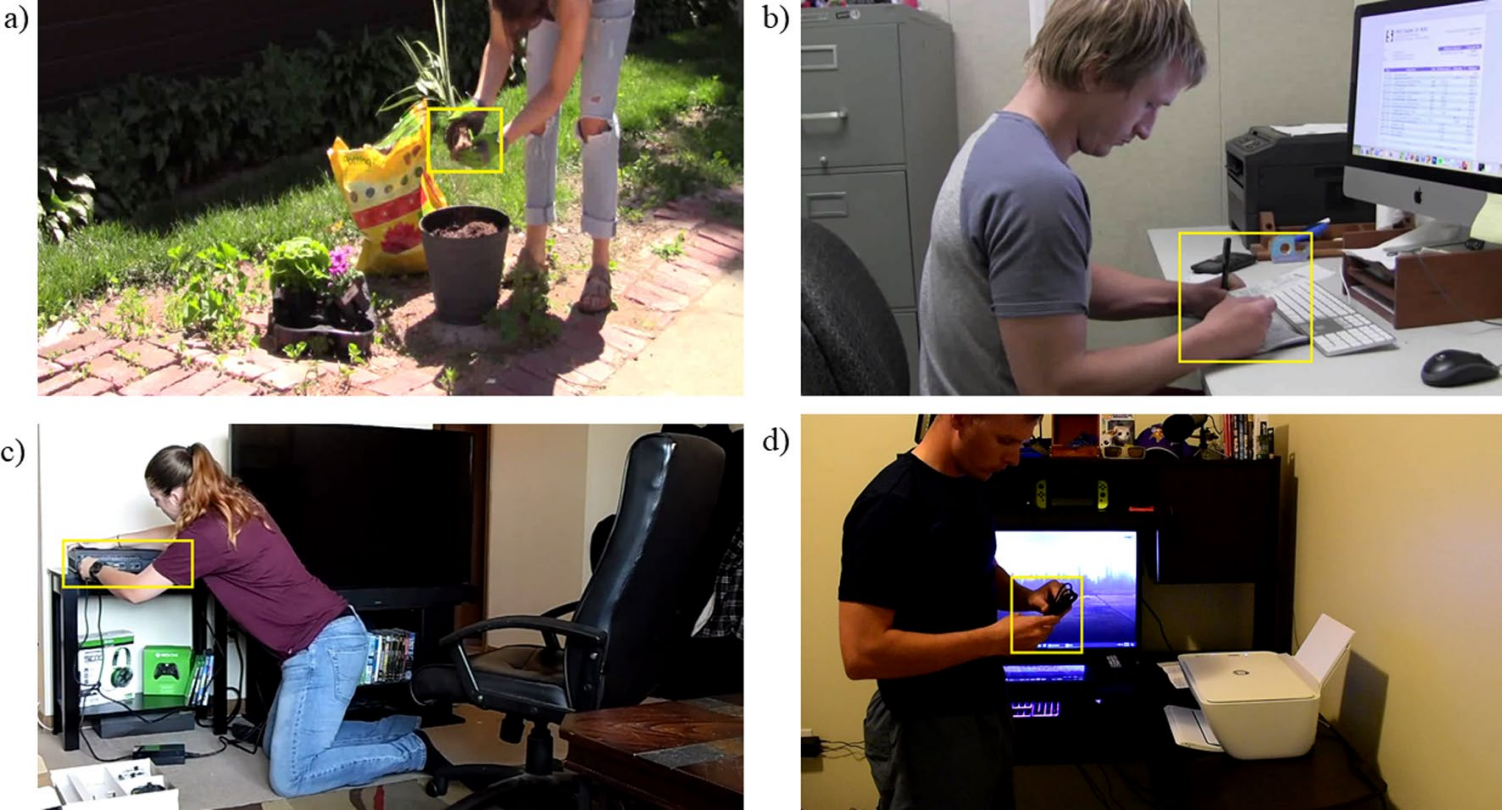
Event stimuli. Stills taken from each of the four experimental movies: **a** planting flowers, **b** balancing a checkbook, **c** setting up a game console, **d** installing a printer. The yellow box represents the interest area, which was drawn around the objects in the focus of the actor’s attention. These objects are critical for completion of the current goal. Participants did not see the yellow box while watching the videos. The size of the interest areas was controlled for in the eye movement analyses

Participants began by watching a practice video of a person building a boat from Duplo’s blocks (duration = 155 s; Zacks et al., 2006) to get familiarized with the procedure and the eye tracker. They then watched four videos that we shot at a rate of 25 frames per second. The movies were filmed from a fixed location without zoom, and they did not have sound. The actors in the videos were all young adults. To test the role of knowledge on attention to goal-relevant content in a film, we needed to find activities where older adults lacked relevant knowledge and are presumably less able to infer the goals of the actors. Four videos depicted activities that vary in familiarity to older and young adults (see Fig. 1). Older adults had more knowledge of two of the videos than young adults (planting a pot of flowers: duration = 297 s; balancing a checkbook: duration = 258 s), and thus, we refer to them as the *older adult activities* (see also Smith et al., 2020). Young adults had more knowledge of the remaining two videos (installing a printer: duration = 148 s; setting up a video game console: duration = 267 s), and we refer to them as *young adult activities*. Two of the videos have been used in prior studies examining the effect of knowledge on event segmentation and memory (Smith et al., 2020).

These activities were selected through a series of pilot studies in our lab using independent samples of older and young adults. In the first pilot study, we asked young and older adults to generate a list of activities for which they were unfamiliar but their age counterparts were familiar with (e.g., “List activities that you are unfamiliar with that younger adults, such as your children or grandchildren, are familiar with.”). Based on their responses, we selected activities that were reported as unfamiliar to older adults (young adults activities: e.g., setting up a printer, backing up a smart phone, setting up a sound system, setting up a video game console) and to young adults (older adults activities: e.g., ironing a shirt, planting flowers, balancing a checkbook, using a record player). Then in the second pilot study, 22 young and 27 older participants were asked to list the sequence of steps involved in the activities selected from the first pilot study. We did not ask participants in the main study to provide scripts for these activities because we did not want the script generation process to contaminate how they attended to the actions in the videos, or the video watching to contaminate the script generation process.

Finally, we selected two young adult activities and two older adult activities that showed strong consensus in the generated scripts and that would be feasible to film. Importantly, we filmed actors performing the activities using the normative scripts provided from the participants. Although participants may have knowledge, to some degree, with all of the activities or the objects within each of the videos, we categorized the activities based on 1) the quality of the scripts produced by each age group (see Smith et al., 2020 for inferential statistics), and (2) the self-reports of knowledge as determined from the two pilot studies reported above.

The videos were shown at a frame rate of 25 frames per second (fps) at a resolution of 1080 × 720 pixels. The video clips were shown on a 17″ ViewSonic Graphics Series CRT monitor (Model G90fb). A chin rest was used to stabilize each participants’ head at a fixed viewing distance of 60.96 cm.

Eye tracking was done using an EyeLink1000 + eye tracker (SR Research), which samples the eye position 1000 times per second (1000 Hz). Based on the guidelines provided by SR Research, an average spatial accuracy of 0.5 degrees of visual angle and a maximum error of 1 degree or better were obtained for all calibrations. Participants were recalibrated to the eye tracker after watching each video.

#### Self-reported familiarity of activities

Participants were asked to report their subjective familiarity with each of the activities at the end of the experiment. When pilot testing this experiment, we found that participants did not use the entire range of the scale when making judgments of their familiarity. As such, we presented participants with a pair of activities (e.g., balancing a checkbook vs. installing a printer) on each trial and were asked to select the activity for which they were more familiar. They completed this process for all 6 pair combinations (4 activities choose 2 activities per trial = 6 trials). To test whether self-reported familiarity aligned with our familiarity manipulation, we performed a mixed effects logistic regression with the participant intercept as a random effect. If an older adult activity was selected, then the response was coded as a 1 and a 0 if a young adult activity was selected. We found that older adults (*M* = 0.98, SE = 0.01) were significantly more likely to indicate they were familiar with performing the older adult activities than young adults (*M* = 0.30, SE = 0.09), *β* = − 4.69, SE = 0.80, *z* = − 5.86, *p* < .001. Thus, older adults selected the older adult activities on 98% of the trials and young adult activities on only 2%, whereas young adults selected the young adult activities on 70% of the trials and the older adult activities on 30% of the trials. Given that 98% of older adults selected the older adult activities in the task, we were unable to use this measure as an individual difference of familiarity.

After making their selection, participants reported how long it had been since they performed each activity, and how often they performed it (Daily, Weekly, Monthly, Yearly, Never). Self-reports for how often older and young adults performed each activity were generally consistent with the notion that older adults had more knowledge of the older adult activities, and that young adults had more knowledge with the young adult activities (see Table 2). For instance, older adults were more likely to report that they never set up a video game console, or that they only do so yearly, whereas many young adults reported doing it daily. Likewise, young adults reported that they have never balanced a checkbook or only do so yearly, whereas many older adults reported that they balance their checkbooks weekly. Compared to the video game and checkbook activities, installing a printer and planting flowers were familiar to both age groups. In the future, researchers may want to consider using different activities or more videos. We examined how self-reported familiarity with each of the activities influenced eye movements and memory in a set of exploratory analyses.

**Table 2:** Self-reported data for how often participants perform each of the activities

	Daily	Weekly	Monthly	Yearly	Never
*Setting up a game console*					
Age group					
Older	0	1	0	1	28
Young	20	1	5	0	7
*Setting up a printer*					
Age group					
Older	0	1	3	18	8
Young	0	1	1	25	4
*Balancing a checkbook*					
Age group					
Older	2	10	11	2	5
Young	0	0	9	9	15
Planting flowers					
Age group					
Older	5	13	6	6	0
Young	0	2	5	19	7

### Goal-relevant areas of interest (AOI)

We placed dynamic interest areas around the object that was the focus of the agents’ attention using DataViewer software (SR Research) (yellow boxes in Fig. 1). To create the AOIs, we first constructed a list of the basic actions performed by the actor using the criteria specified in the Action Coding System (ACS), termed A1 units (Schwartz et al., 1991). The ACS constructs goal hierarchies of action sequences. A1 units are the basic actions involved in completing higher-level goals, such as “connect the power cable into the power supply box” and “insert the power supply into the xbox’s power port” before “plugging the xbox into the wall outlet”. A2 units are the higher-level goals such as “connect the xbox to a power outlet”. Our ACS coding for each of these videos is available on the Open Science Foundation (OSF) website associated with this manuscript (https://osf.io/jkxn4/). In each frame of each video, we placed an AOI around the critical object important for completing each basic A1-level goal. Thus, the AOIs changed location as the video progressed. AOIs were placed using the following rules: (1) all AOIs were rectangular in shape; (2) no AOIs were allowed to overlap in time or space; (3) AOIs contained the information critical to performing the current goal of the actor in the video, which we identified from the ACS, and (4) AOIs changed when a goal was completed. It is important to note here that the size of the AOIs differed based on the goal-related object (e.g., TV versus remote control). We could predict that a larger size AOI is more likely to be fixated due to random chance than a smaller AOI. Thus, AOI size was controlled in the eye movement analyses, where it could potentially have an impact on the results, but not in the memory analyses, where such a relationship is not obvious, using a simultaneous multiple regression.

#### Recall test

After completing a psychometric filler task, participants were given 5 min to recall what had happened in the video, in as much detail as possible, to the best of their ability. We used the ACS to score the free recall responses (Schwartz et al., 1991). First, two coders, blind to the age group of the participant, coded recall data for each video for 10 participants. They began by counting the number of A1 units (fine-grained goals) and A2 units (coarse grained goals) each participant successfully recalled. Videos differed in the number of goals performed by the actor. There were 84 A1 and 27 A2 units in the checkbook video, 94 A1 and 23 A2 units in Planting flowers, 90 A1 and 26 A2 units in the setting up a video game console video, and 56 A1 and 35 A2 units in the installing a printer video. Raters produced strong inter-rater reliability (Cohen’s Kappa = 0.79, *p* < .05; for A1 units, Cohen’s Kappa = 0.87, *p* < .05 for A2 units). Discrepancies between the two coders were resolved through thoughtful discussion. They then independently scored data from the remaining participants blind to the participants’ age group. Each rater scored half of the data (approximately 25 participants) from each video. The proportion of correctly recalled A1 and A2 units was used as the dependent measure.

#### Recognition test

**Fig. 2:**
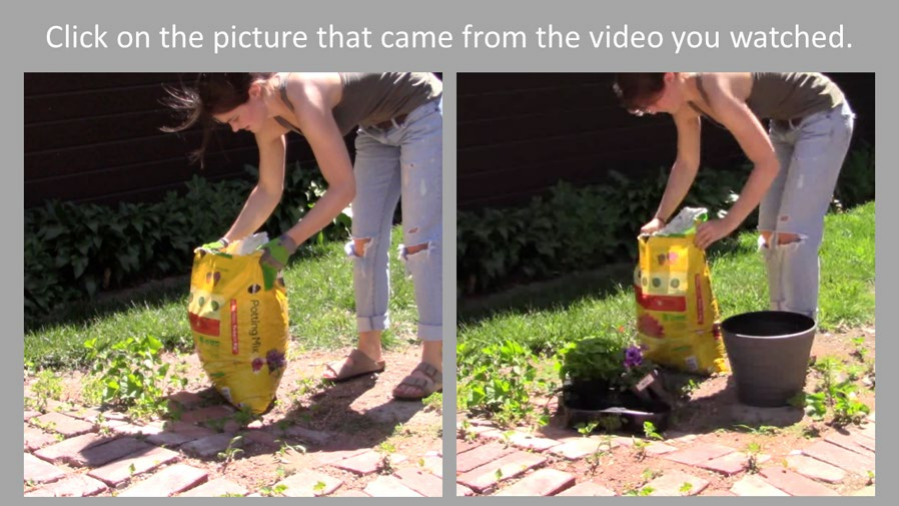
An example recognition memory test trial. Participants were asked to select the picture that came from the video they watched. The target image is on the right, and the distractor image is on the left in this example

Recognition memory was assessed using a two-alternative forced choice task (see Fig. 2). There were 20 total recognition memory trials. Pairs of images were shown on each trial side by side. Target images were frames taken from the videos that participants watched. Distractor items on each trial were taken from a different video of the same actor performing the same action (e.g., Planting a pot of flowers), but in a different order (e.g., the actor set the potting soil on the ground after bringing in the flowers and planter in the target video, but set the potting soil on the ground before the pot in the foil video), or involving different actions (e.g., the actor planted petunias in the target video, but not in the foil). Participants were asked to select the image that came from the video they watched. The pairs of target images and foils were randomized across participants, and the correct answer was counterbalanced (left versus right) within each participant. The recognition memory measure was internally consistent, as evident from acceptable Cronbach’s alpha values across videos (*α* = 0.76 planting flowers; 0.86 balancing a checkbook; 0.64 setting up a video game console, 0.71 installing a printer). Recognition performance was scored as the proportion of correctly identified trials.

#### Order test

Order memory was also assessed using a two-alternative forced choice task. Like the recognition memory test, pairs of images were presented on each trial. Twelve images were taken from the videos that participants watched. Each picture was paired with each of the other images within the set. Each pair of images were presented once for a total of 66 order memory trials (12 images choose 2 images = 66 trials). Participants were instructed to indicate the action that occurred first in the video. The next pair appeared after participants responded. The order memory measure was also internally consistent as evident from acceptable Cronbach’s alpha values across videos (*α* = 0.83 Planting flowers; 0.88 Balancing a checkbook; 0.76 Setting up a video game console, 0.84; Installing a printer). Order memory performance was scored as the proportion of correctly identified trials.

### Procedure

Participants first completed the MMSE. Then, participants’ eyes were calibrated on the eye tracker. They then watched the practice video to get comfortable with the eye tracker. After watching the practice video, participants watched the first experimental video. All videos were shown without sound and without a title. Prior work has shown that providing titles can activate relevant schema, which influences encoding and retrieval (Bransford & Johnson, 1972; Miller & Stine-Morrow, 1998; Newberry & Bailey, 2019), and we only wanted the visual content in the video to activate relevant knowledge (like experiencing events in the real world).

After each video, participants completed a psychometric test that served as a 5-min distraction task. Psychometric tests included tests of processing speed, vocabulary, script generation, event segmentation ability, and working memory capacity. Details of each measure are reported in the Additional file 1, and descriptive statistics for each of these measures for older and young adults are shown in Table 1. Participants had typical scores on these psychometric tests. The order of these distractor tasks was not counterbalanced across participants, but we did counterbalance the order of the videos.

Participants completed the three event memory measures discussed above (free recall, recognition and then order memory, in that order, to avoid contaminating the recall by the recognition and order memory measures) after the 5-min distractor task. Participants then repeated the following steps for the remaining 3 videos: watch a video, take a psychometric test, and take memory tests. After completing the final memory test for the final video, participants segmented the videos (see Additional file 1 for details about this task) and then reported their familiarity with each of the activities. Finally, the participants were debriefed and thanked for their participation.

## Results

We first report the eye movement and attention to goal-relevant AOIs results. These analyses are followed by the memory results. Lastly, we report how selective attention to goal-relevant information in the videos predicted event memory. To foreshadow our findings, we found an age-related deficit in both attention to goal-relevant information and event memory when older adults watched the unfamiliar activities, but we found no age-related differences when older adults watched the familiar activities. In addition, we found that attention to goal-relevant information predicted free recall memory, but only when participants were familiar with the activities.

We conducted all analyses in R (version 4.0.0). Linear mixed models were run using the lme4 package (Bates, Mächler, Bolker, & Walker, 2014). In all of the analyses, age group and activity type were effect coded as Young = − 1 and Older =  + 1 before they were entered into the mixed models. The afex package was used to estimate *p* values of fixed effects (Luke, 2017; Singmann et al., 2015). We used the anova() function from the lmerTest package to estimate *F* and *p* values of predictors with more than two levels (Kuznetsova et al., 2017). We derived *F* values using type 3 sums of squares. Estimated least square means and their corresponding standard errors were fit using the emmeans function in the emmeans package (Lenth, 2016). Degrees of freedom were corrected using a Kenward–Rogers approximation (Kenward & Roger, 1997). All significant interactions were probed using the emmeans package. Interactions were probed using planned contrasts, which were corrected with a Bonferroni adjustment. A Tukey adjustment was applied to exploratory analyses.

### Attention to goal-relevant information

**Fig. 3:**
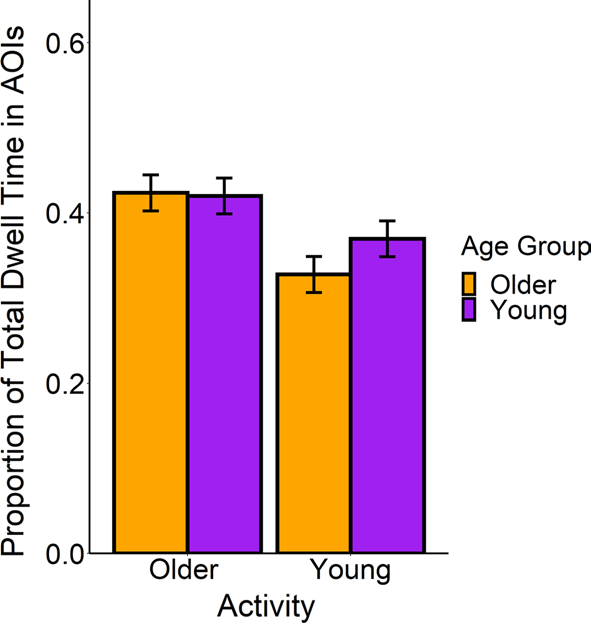
Proportion of total dwell time spent in the goal-relevant AOIs. Young adults spent more time in the goal-relevant regions in the young adult activities, but older and young adults did not differ in their time spent looking at goal-relevant information in the older adult activities. Error bars represent 1 standard error around the estimated means

In our first analysis, we evaluated how knowledge affected attention to goal-relevant information when young and older adults were more versus less familiar with the activity. See Fig. 3. We removed three older adults and two young adults from the eye tracking analysis because we were unable to calibrate them accurately on the eye tracker. We then fit a linear mixed model to the proportion of time participants spent looking within the areas of interest. To calculate this proportion, we took the ratio between the total dwell time in the AOIs for each participant and divided it by the total amount of dwell time recorded for that participant. This was to account for differences in the number of fixations acquired from each participant. The model included the age group (young = − 1 vs. older =  + 1), the type of activity (young adult activity = − 1 vs. older adult activity =  + 1), and their interaction as fixed effects. Further, we treated the standardized average size of the AOIs as a continuous additive covariate in the model as a fixed effect. The additive covariate was included in all of the models that contained eye movement measures as the dependent measure. We assumed that people would be more likely to attend to the AOI when it was large, so we took that into account in the analyses.

To determine the random effect structure, we fit three different models with the same fixed effects mentioned above and varied only the random effects between models. We evaluated model fitness by comparing the models with different random effect structures with a likelihood-ratio test (Bates et al., 2014; Matuschek et al., 2017). We started with the “maximal model” (Barr et al., 2013) and then reduced it by removing only a single random effect until the more complex model was a statistically better fit of the data than the reduced model. We retained the more parsimonious model when it did not significantly differ from the more complex model. Participant and video were both treated at their intercept as random effects in all of the models to decorrelate the effects contributed by each subject with the manipulation and to account for by-item mean differences (Singmann & Kellen, 2019).

The first model was the maximal model in which the effect of age group was allowed to vary for each video as a random effect (e.g., the by-item random intercept and by-item random slopes), and the effect of activity type was allowed to vary for each participant as a random effect (e.g., the by-participant random intercept and by-participant random slopes). We removed the age group slope as a random effect in the second model, preserving the by-item intercept and the by-participant random intercept and slope of activity type. We removed the activity type slope as a random slope effect in the third model, so that the third model only contained the by-participant and by-item intercepts as random effects. The first and second model did not significantly differ, *χ*^2^(2) = 0.08, *p* = .96, suggesting that the by-item random slope of age group was unnecessary, and neither did the second and third model, *χ*^2^(2) = 2 . 93, *p* = .23, which suggests that the by-participant random slope of activity type was also unnecessary to improve the model fit. Thus, we retained and reported the results of the third model, which only included the participant and video at their intercept as random effects.

As shown in Fig. 3, we found that older adults attended less to goal-relevant information than young adults when they watched the young adult videos, but not when they watched the older adult videos. The standardized average size of the AOI did not significantly predict the time spent looking at the goal-relevant information, *β* = 0.08, SE = 0.03, *t* = 2.22, *p* = .27. Further, we did not observe a significant difference in the proportion of time spent looking at the AOIs between age groups [Young Adults (*M* = 0.40, SE = 0.02); Older Adults (*M* = 0.38, SE = 0.02)], *β* = − 0.01, SE = 0.007, *t* = − 1.29, *p* = .20, *d* = 0.11,^1^ or the type of activity [Young Adults Activities (*M* = 0.35, SE = 0.04); Older Adults Activities (*M* = 0.42, SE = 0.04)], *β* = 0.04, SE = 0.03, *t* = 1.05, *p* = .48, *d* = 0.42. However, we did observe a significant age group by activity type interaction, *β* = 0.01, SE = 0.004, *t* = 2.83, *p* = .005, *d* = 0.13, such that there was an age-related deficit in the amount of time spent in the goal-relevant areas of interest when older adults lacked prior knowledge for the activity, but this deficit disappeared when older adults possessed relevant prior knowledge. This is evident in Fig. 3. Compared to young adults (*M* = 0.37, SE = 0.04), older adults (*M* = 0.33, SE = 0.04) spent less time looking at the AOIs in the young adult activities, *β* = − 0.04, SE = 0.02, *t* = − 2.48, *p* = .03. However, older (*M* = 0.42, SE = 0.04) and young adults (*M* = 0.42, SE = 0.04) did not significantly differ in the amount of time looking at the AOIs in the older adult activities, *β* = 0.004, SE = 0.02, *t* = 0.22, *p* = .99. Thus, older adults were less likely to attend to important goal-relevant information when they lacked relevant knowledge, but showed similar patterns of goal-related eye movements as the young adults when they could rely upon prior knowledge. This is among the first experiments to demonstrate older adults’ comprehension, as informed by prior knowledge, may influence where they look in videos of everyday activities. Analogous findings were observed when we used the proportion of fixations, rather than the proportion of time, in the AOIs as the dependent measure. See the Additional file 1 for those results.

### Exploratory analysis

#### The effects of stimuli on attention to goal-relevant information

We also ran the analysis using the video as a fixed effect to explore possible differences in attention to goal-relevant information between videos. Perhaps the results were primarily driven by the two videos (balancing a checkbook and setting up a video game console) that showed the strongest familiarity differences between age groups. To foreshadow our results, we indeed found the largest age-related attentional difference in the *Video game console* video. To address this question, we conducted an exploratory analysis in which we entered the age group, video, and their interaction into a linear mixed effects model of the proportion of time spent in the AOI. The by-participant intercept was included as a random effect. We did not observe a significant effect for age group, *F*(1,55) = 1.65, *p* = .20, but we did for video, *F*(3, 165) = 68.05, *p* < .001. Further, older and young adults’ attention to goal-relevant information differed between videos, *F*(3,165) = 3.88, *p* = .01. We probed this interaction and found that older and young adults did not significantly differ in time spent looking in the goal-relevant areas of interest in the checkbook [Young Adults (*M* = 0.36, SE = 0.01); Older Adults (*M* = 0.36, SE = 0.01)], *β* = − 0.004, SE = 0.02, *t* = − 0.21, *p* = .99; gardening [Young Adults (*M* = 0.36, SE = 0.01); Older Adults (*M* = 0.37, SE = 0.01)], *β* = 0.01, SE = 0.02, *t* = 0.57, *p* = .99; or printer [Young Adults (*M* = 0.35, SE = 0.01); Older Adults (*M* = 0.33, SE = 0.01)], *β* = − 0.02, SE = 0.02, *t* = − 1.09, *p* = .99 videos; however, young adults attended more to goal-relevant information in the setting up a video game console video [Young Adults (*M* = 0.51, SE = 0.01); Older Adults (*M* = 0.45, SE = 0.01)], *β* = − 0.06, SE = 0.02, *t* = − 3.03, *p* = .01 (all *p* values were adjusted using a Bonferroni correction). In line with the self-report data, young adults showed the largest advantage in attention to goal-relevant information for the setting up a video game console activity though time spent in the goal-relevant areas was numerically higher for the printer video.

### The effects of self-reported familiarity on attention to goal-relevant information

Differences in the proportion of time spent in the goal-relevant areas of interest suggests that attention to goal-relevant information could depend upon one’s self-reported familiarity with the activities. As such, we explored if self-reported familiarity performing the activities influenced attention to goal-relevant information in an exploratory analysis. Unfortunately, we were unable to cross the activity type predictor with how often participants reported that they perform each activity (Daily, Weekly, Monthly, Yearly, and Never) as an interaction effect. This was because we lacked a sufficient number of observations for many combinations of each video and self-reported familiarity with the activity (see Table 2). As such, the model only contained the continuous covariate of AOI size, the main effect of age group, self-reported familiarity (Daily, Weekly, Monthly, Yearly, and Never), and their interaction as fixed effects. We compared two models to determine the random effect structure of the model we retained. Both models contained the participant and video intercepts as random effects. We allowed the age group of the participants to vary as a random slope effect with the video in the first model. We did not in the second. The two models did not significantly differ, so we retained the second model, *χ*^2^(2) = 0.001, *p* = .99, which included the participant and video at their intercept as random effects.

**Fig. 4:**
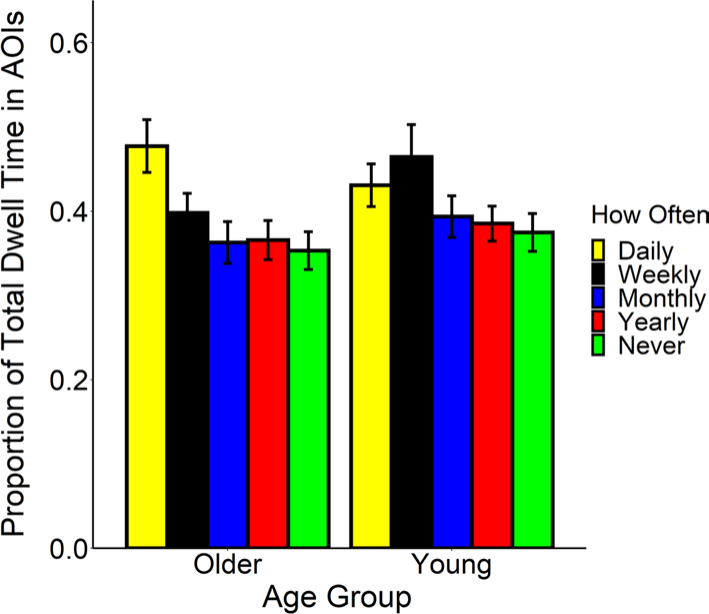
Proportion of total dwell time spent in the goal-relevant areas of interest. Participants dwelled more in the goal-relevant regions when they performed the activity daily than if they performed the activity monthly, yearly, or never. Error bars represent 1 standard error around the estimated means

As shown in Fig. 4, the proportion of time spent looking in the goal-relevant areas of interest depended on how often participants performed the activity. Both young and older adults were more likely to attend to goal-relevant information if they regularly performed the activity. Again, the average size of the AOI did not significantly predict the time spent looking at the goal-relevant information, *F*(1, 2.22) = 7.77, *p* = .10. We also did not observe a main effect for age group, *F*(1, 81.52) = 1.36, *p* = .25, but we did for self-reported familiarity, *F*(4,195.07) = 8.99, *p* < .001. We compared attention to goal-relevant information for each of the levels of familiarity performing the activities. As shown in Fig. 4, we probed the main effect of self-reported familiarity and found that participants dwelled longer in the AOIs if they performed the activity daily (*M* = 0.45, SE = 0.03) compared to monthly (*M* = 0.38, SE = 0.03), *β* = 0.08, SE = 0.02,* t* = 3.81, *p* = .002, daily compared to yearly (*M* = 0.37, SE = 0.03), *β* = 0.08, SE = 0.02, *t* = 4.20, *p* = .0004, and daily compared to if they reported they never performed the activity (*M* = 0.36, SE = 0.03), *β* = 0.09, SE = 0.02, *t* = 5.46, *p* < .0001. They also dwelled longer in the areas of interest if they reported that they performed the activity weekly (*M* = 0.43, SE = 0.03) as compared to when they reported they never performed the activity, *β* = 0.07, SE = 0.02, *t* = 3.25, *p* = .01. None of the other differences were significant (all *p* values > 0.05 after the Tukey adjustment). Thus, individual differences in familiarity with the activities influenced the extent to which participants attended to goal-relevant information in the videos. Finally, our results suggest that both older and young adults’ attention to goal-relevant information was influenced to the same degree by self-reported familiarity with the actions, because the interaction between age and self-reported familiarity was not significant, *F*(4,192.73) = 1.60, *p* = .18. Even young adults show a deficit in attention to goal-relevant information if they rarely or never perform the activity.

### Memory

Next, we evaluated age- and knowledge-related differences on event memory performance. The mean performance on the memory measures as well as correlations between these measures for all participants are given in Table 3. Performance on all three memory measures was moderately to strongly correlated (range 0.26–0.81). As such, we first standardized performance across participants and videos on each of the memory measures as Z-scores using the separate means and standard deviations for each measure. We then calculated an average composite memory score for each participant and video by averaging the Z-scores for each measure. We fit a linear mixed model to this average memory score. Age group, activity type, and their interaction were treated as fixed effects. We also ran the analyses separately for each memory measure. Results did not differ between the different types of memory. See the Additional file 1 for details of those analyses.

**Table 3:** Memory performance and Pearson correlations of each of the different memory measures

	*M*	SE	Recognition	Order memory	Proportion of A1s recalled	Proportion of A2s recalled
Recognition	0.66	0.01	–			
Order memory	0.86	0.01	0.48	–		
Proportion of A1s recalled	0.15	0.005	0.26	0.36	–	
Proportion of A2s recalled	0.42	0.02	0.34	0.49	0.81	–

All correlations were significant, *p* < .0001

We used the same method as we reported in the AOI analysis to determine the random effect structure of the model we retained. We compared three models using a likelihood-ratio test to evaluate if a simpler model was a significantly worse model than the fit of the more complex model as before using the average event memory as the dependent measure. The maximal model did not significantly differ from a model that contained the by-participant random slope of activity type and the by-item intercept of video, *χ*^2^(2) = 8.83, *p* = .07; nor did it differ from a model that included only the participant and video intercepts as random effects. Therefore, we retained the more parsimonious model, *χ*^2^(2) = 0.45, *p* = .79.

**Fig. 5:**
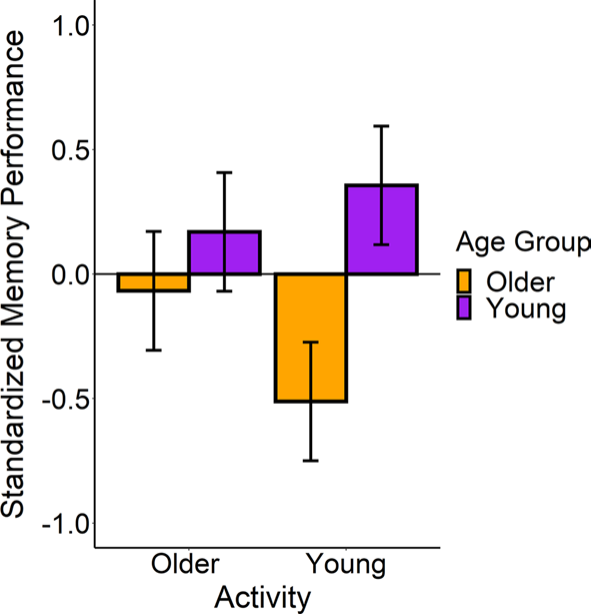
Event memory performance as a function of activity and age group. Young adults had significantly better memory for the young adult activities, but older and young adults did not differ in their memory performance for content in the older adult activities. Error bars represent 1 standard error around the estimated means

Figure 5 shows that we found a similar pattern of results to what we observed with the amount of dwell time spent in the areas of interest. In sum, we found an age-related deficit in memory performance between age groups only when the activities were unfamiliar to older adults. We did not observe a significant main effect for the activity type on memory performance [Young Adult Activities (*M* = − 0.08, SE = 0.41); Older Adult Activities (*M* = 0.05, SE = 0.41)], *β* = 0.06, SE = 0.33, *t* = 0.19, *p* = .86, *d* = 0.08. As expected, we observed a significant main effect of age group such that young adults (*M* = 0.28, SE = 0.30) had significantly better memory than older adults (*M* = − 0.31, SE = 0.30), *β* = − 0.28, SE = 0.05, *t* = − 5.11, *p* < .001, *d* = 0.32. However, Fig. 5 also shows that this main effect was qualified by a significant interaction between the age group and the activity type, *β* = 0.16, SE = 0.02, *t* = 6.66, *p* < .001, *d* = 0.18. When we probed this interaction, we found that age-related deficits in episodic memory arose when older adults lacked the relevant knowledge of the activity. Specifically, we found that young adults’ memory performance (*M* = 0.37, SE = 0.42) was almost one standard deviation greater than that of the older adults (*M* = − 0.53, SE = 0.42) in the young adult videos, *β* = − 0.87, SE = 0.12, *t* = 7.36, *p* < .001; however, young and older adults did not significantly differ in their memory of the older adult videos [Young Adult (*M* = 0.18, SE = 0.42); Older Adult (*M* = − 0.08, SE = 0.42)], *β* = − 0.24, SE = 0.12, *t* = − 2.00, *p* = .10. Thus, like the eye movement results, older adults only showed a deficit in their memory performance when they had less knowledge of the activities. We observed analogous effects with each of the separate memory measures when we ran the analyses separately for each measure (see the Additional file 1 for details).

### Exploratory analysis

#### The effects of stimuli on memory

It is possible that the memory differences we observed were driven by only a subset of the videos. To evaluate this, we ran the same analysis as above but included the video as a fixed effect. We entered the age group, video, and their interaction into a linear mixed effects model of the average standardized event memory. Participant was included as a random effect. We found the largest age-related difference in memory for the *Video game console* video. Overall, older adults (*M* = − 0.29, SE = 0.08) had significantly worse memory than young adults (*M* = 0.26, SE = 0.07), *F*(1,61) = 26.13, *p* < .001. We observed a significant main effect for video, *F*(3, 183) = 140.00, *p* < .001, as well as a significant interaction between video and the age group, *F*(3, 183) = 20.05, *p* < .001. We probed this interaction and found that older and young adults did not significantly differ in memory for the checkbook video [Young Adults (*M* = − 0.57, SE = 0.10); Older Adults (*M* = − 0.65, SE = 0.10)], *β* = − 0.07, SE = 0.13, *t* = − 0.53, *p* = .99, but young adults had significantly better memory in the gardening [Young Adults (*M* = 0.91, SE = 0.10); Older Adults (*M* = 0.51, SE = 0.10)], *β* = − 0.40, SE = 0.13, *t* = − 2.99, *p* = .01; printer [Young Adults (*M* = 0.25, SE = 0.10); Older Adults (*M* = − 0.45, SE = 0.10)], *β* = − 0.70, SE = 0.13, *t* = − 5.21, *p* < .001, and the video game console video [Young Adults (*M* = 0.47, SE = 0.10); Older Adults (*M* = − 0.57, SE = 0.10)], *β* = − 1.04, SE = 0.13, *t* = − 7.72, *p* < .001 (Bonferroni adjusted *p* values).

#### The effects of self-reported familiarity on event memory

**Fig. 6:**
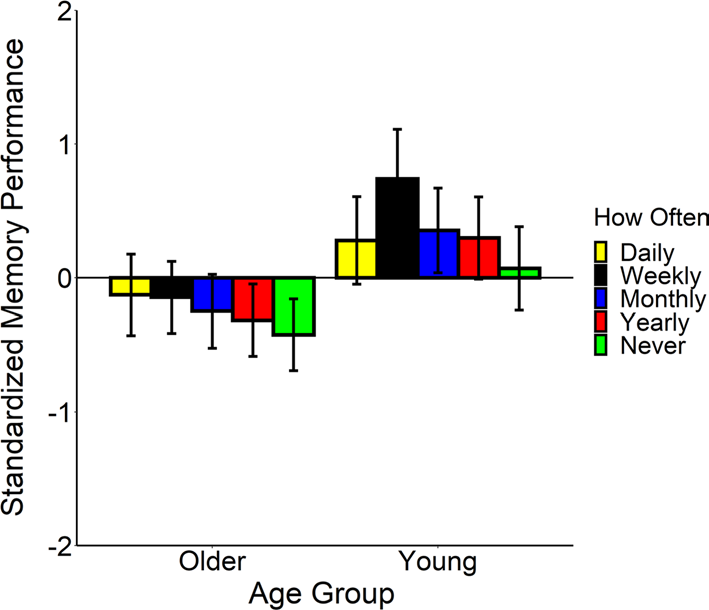
Average event memory performance as a function of how often older and young adults performed the activity. Participants remembered more when they performed the activity daily than if they performed the activity monthly, yearly, or never

We also explored if activities performed often by the participants were remembered better than activities that are performed rarely or never at all. As shown in Fig. 6, young adults showed the typical memory advantage, but both age groups had better memory of the activities that they performed more often, consistent with the hypothesis that knowledge influenced participants’ memory for the activities. The linear mixed effects model contained the age group, self-reported familiarity with each activity, and their interaction as fixed effects of participants’ event memory. As we did in the evaluation of self-reported familiarity and eye movements, we compared two models to determine the random effect structure. Both contained the participant and video intercepts as random effects. We also allowed the age group to vary as a random slope effect with video in the first model, but we did not in the second model. The models differed significantly, so we retained the first model, *χ*^2^(2) = 10.39, *p* = .006.

Again, young adults had (*M* = 0.35, SE = 0.31) significantly better memory than older adults (*M* = − 0.25, SE = 0.26), *F*(1, 5.28) = 9.57, *p* = .02. Like the eye movement results, we also observed a significant main effect for self-reported knowledge with the activities, *F*(4, 198.14) = 3.44, *p* = .01. We compared each level of self-reported knowledge using a Tukey adjustment for the *p* values. As shown in Fig. 6, participants had significantly better memory if they have ever performed the activity than if they never perform the activity [Daily (*M* = 0.08, SE = 0.29) compared to never (*M* = − 0.18, SE = 0.27), *β* = 0.25, SE = 0.13, *t* = 1.93, *p* = .04; Weekly (*M* = 0.30, SE = 0.29) compared to never, *β* = − 0.47, SE = 0.14, *t* = − 3.46, *p* = .0007; Monthly (*M* = 0.05, SE = 0.27) compared to never, *β* = 0.23, SE = 0.10, *t* = 2.39, *p* = .02; Yearly (*M* = − 0.008, SE = 0.27) compared to never, *β* = − 0.17, SE = 0.08, *t* = − 2.05, *p* = .04]. They also had significantly better event memory if they performed the activity weekly than yearly, *β* = 0.31, SE = 0.13, *t* = 2.30, *p* = .02. Again, our data suggest that older and young adults demonstrated the same memory benefit due to their knowledge of the activities because we did not observe a significant interaction between age and how often the participants performed each activity, *F*(4, 199.80) = 0.79, *p* = .53, so even young adults showed an advantage in event memory if they performed the activity often.

### Effects of attending to goal-relevant information on memory

**Fig. 7:**
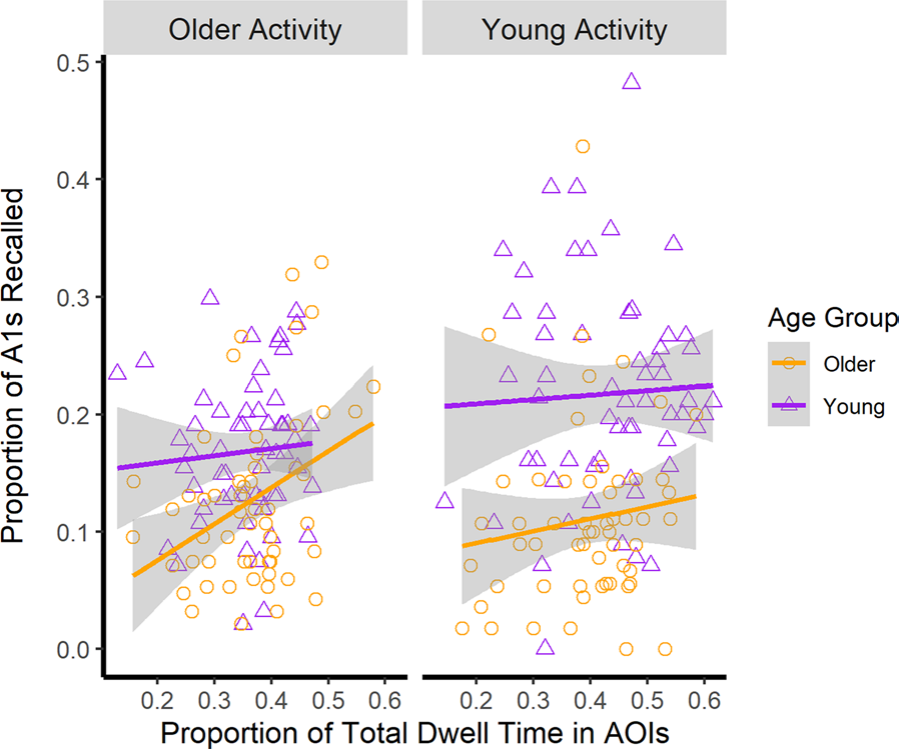
Proportion of A1s recalled as a function of the proportion of time spent in the AOIs, activity, and age group. Raw values for each participant are represented by the points in the figure. Activity type is represented in the panels. Lines represent the predicted least square means generated from the estimated regression equation. The proportion of total dwell time in the areas of interest positively predicted event memory. Error ribbons correspond to 1 standard error around the line of best fit

**Fig. 8:**
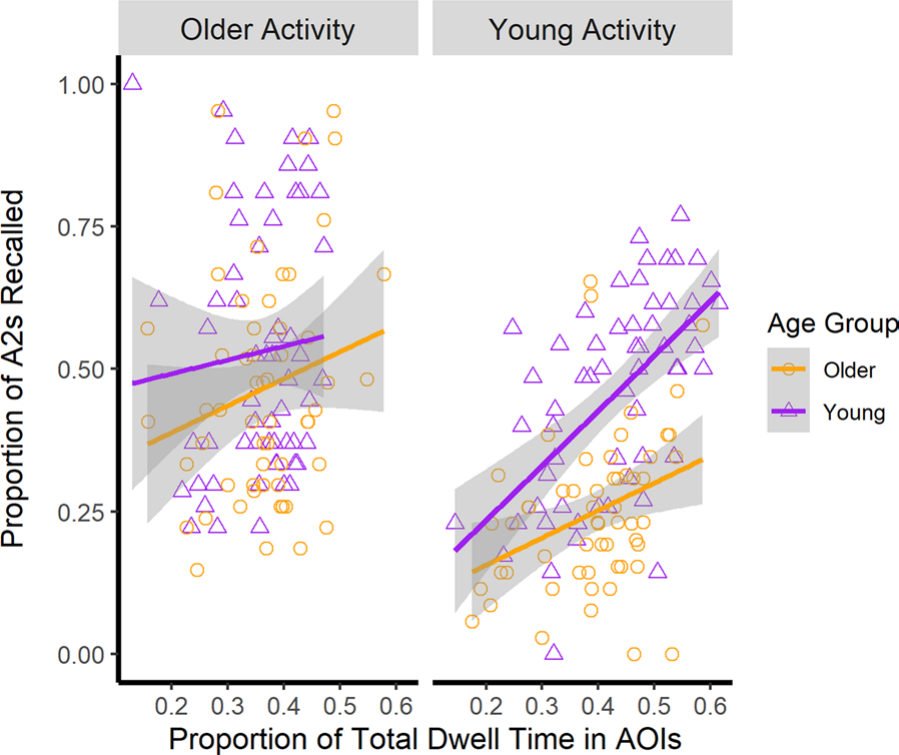
Proportion of A2s recalled as a function of the proportion of time spent in the AOIs, activity, and age group. Raw values for each participant are represented by the points in the figure. Activity type is represented in the panels. Lines represent the predicted least square means generated from the estimated regression equation. The proportion of total dwell time in the areas of interest positively predicted event memory. Error ribbons correspond to 1 standard error around the line of best fit

Lastly, we evaluated whether attention to goal-relevant information produced better event memory. Given that maintenance of goal related information is important for successful comprehension, we evaluated whether individual differences in the time spent in the AOIs predicted participants’ memory for the videos. As shown in Figs. 7 and 8, we found that the percent of time spent in the goal-relevant areas of interest positively predicted free recall memory; however, it did not produce better recognition or order memory (see Table 4).

**Table 4:** Table of fixed effects for recognition, order, and free recall memory performance as a function of age group, activity type, and proportion of dwell time in the goal-relevant AOIs

Fixed effect	*β*	SE	*t*	*p*
*Proportion of A1 units recalled*				
Intercept	0.16	0.01	30.04	< .001***
Age group	− 0.04	0.01	− 6.85	< .001***
Activity type	− 0.01	0.01	− 1.06	.29
Proportion of dwell time	0.13	0.06	2.28	.02*
Age group*activity type	0.02	0.01	3.32	< .01**
Age group*proportion of dwell time	0.08	0.06	1.40	.16
Activity type*proportion of dwell time	0.06	0.06	1.02	.31
Age group*activity type*proportion of dwell time	0.05	0.06	0.81	.42
*Proportion of A2 units recalled*				
Intercept	0.42	0.08	5.29	.03*
Age group	− 0.06	0.01	− 4.33	< .001***
Activity type	0.08	0.08	1.04	.41
Proportion of dwell time	0.25	0.12	2.00	.04*
Age group*activity type	0.03	0.01	3.67	< .001***
Age group*proportion of dwell time	− 0.15	0.10	− 1.47	.14
Activity type*proportion of dwell time	− 0.02	0.10	− 0.18	.86
Age group*activity type*proportion of dwell time	0.12	0.09	1.31	.19
*Proportion correct recognition memory*				
Intercept	0.66	0.06	10.27	.01**
Age group	− 0.03	0.01	− 2.35	.02*
Activity type	0.03	0.06	0.43	.71
Proportion of dwell time	0.00	0.12	− 0.01	.99
Age group*activity type	0.01	0.01	1.36	.18
Age group*proportion of dwell time	− 0.12	0.10	− 1.17	.24
Activity type*proportion of dwell time	0.10	0.11	0.98	.33
Age group*activity type*proportion of dwell time	0.05	0.09	0.60	.55
*Proportion correct order memory*				
Intercept	0.86	0.05	19.07	< .01**
Age group	− 0.03	0.01	− 4.46	< .001***
Activity type	0.00	0.04	0.01	.99
Proportion of dwell time	0.05	0.08	0.70	.49
Age group*activity type	0.01	0.01	1.83	.07
Age group*proportion of dwell time	− 0.03	0.07	− 0.51	.61
Activity type*proportion of dwell time	− 0.07	0.06	− 1.03	.31
Age group*activity type*proportion of dwell time	− 0.01	0.05	− 0.16	.87

Age group and activity type were effect coded as Young = − 1 and Older =  + 1 before entry into the mixed model as fixed effects. We centered the proportion of dwell time in the areas of interest at its mean prior to entry into the analysis

*p < .05; **p < .01; ***p < .001

The extent to which the proportion of time spent in the AOIs predicted memory differed for each memory measure, so we have presented the data from each measure separately. First, we evaluated recall of smaller action units (A1 units), then recall of larger action units (A2 units), followed by recognition and finally order memory. Each model contained the main effect of age group (effect coded as Young = − 1 and Older =  + 1), activity type (effect coded as Young Activity = − 1 and Older Activity =  + 1), the percent of time in the areas of interest (centered at its mean), and their interactions as fixed effects.^2^ We determined the random effect structure we retained by comparing six different models for each dependent measure starting with the maximal model if the model converged. We then reduced it until the by-participant and by-item intercepts were the only two random effects included in the model. We compared models using a likelihood-ratio test as before. The model we retained contained the by-participant and the by-item intercepts as random effects.

#### Recall of A1 units

The model contained the participant and video at their intercepts as random effects. As shown in Fig. 7, those who spent more time fixating the AOI recalled more A1 level goals than those that did not, *β* = 0.13, SE = 0.06, *t* = 2.28, *p* = .02. Since none of the interactions with the proportion of time spent in the AOIs were significant (all *p* values > 0.05; Table 4), this could suggest that attention to goal-relevant information produced better recall of A1 units, for both young and older adults, regardless of their familiarity with the activities. Alternatively, we may not have had sufficient power to detect interactions with proportion of time spent in the areas of interest. The remaining significant effects in the model were consistent with the event memory results reported earlier. There was a main effect for the age group and an interaction between age group and activity type.

#### Recall of A2 units

The model contained the participant and video at their intercept as random effects. We also allowed the effect of activity type to vary for each participant as a random effect. Consistent with recall of A1 units and given in Fig. 8, we found that the percent of time spent looking at the AOIs positively predicted the proportion of A2 units successfully recalled, *β* = 0.25, SE = 0.12, *t* = 2.00, *p* = .04. Again, none of the interactions with the proportion of time spent in the areas of interest were significant (all *p* values > 0.05; Table 4). As such, this could suggest that attention to goal-relevant information produced better recall of A2 goals, for both young and older adults, regardless of their familiarity with the activities.

#### Recognition memory

Participant and video were treated at their intercepts as random effects. As shown in Table 4, we found a significant main effect for age group such that young adults (*M* = 0.69, SE = 0.07) had significantly better recognition memory than older adults (*M* = 0.63, SE = 0.07), *β* = − 0.03, SE = 0.01, *t* = − 2.35, *p* = .02. None of the remaining effects were statistically significant. See Table 4 for details.

#### Order memory

The model contained the participant and video intercepts as random effects. We also allowed the effect of activity type and the proportion of dwell time in the areas of interest to vary as by-participant random slope effects. Consistent with the aforementioned results of average event memory, we found that young adults (*M* = 0.89, SE = 0.05) had significantly better order memory than older adults (*M* = 0.82, SE = 0.05), *β* = − 0.03, SE = 0.01, *t* = − 4.46, *p* < .001. We also observed a marginally significant interaction between age group and activity type, *β* = 0.01, SE = 0.01, *t* = 1.83, *p* = .07. None of the other main effects or interactions were significant for order memory. See Table 4 for details.

## Discussion

Theory and intuition suggest that prior knowledge guides our attention and memory for new relevant information. Consistent with this idea, there is strong evidence for top-down effects on attention when people view static images (e.g., Henderson et al., 2009) or read text (Rayner, 1998). Yet, prior work has largely failed to find strong evidence that one’s event model for a film influences where and for how long we attend, a phenomenon known as *the Tyranny of Film* (Hutson et al., 2017; Loschky et al., 2015)*.* Because our study used short films and found that viewers’ knowledge of the filmed events guided their attention, this study does not provide evidence of the Tyranny of Film (see also, Eisenberg et al., 2018; Flanagan & Johansson, 2003; Hayhoe et al., 2012; Rotman et al., 2006; Tatler et al., 2013).

We had a group of older and young adults watch videos of actors performing everyday activities that were more familiar to older adults (i.e., balancing a checkbook, planting flowers) or young adults (i.e., setting up a video game console, installing a printer). We hypothesized that participants would have richer event models and motor representations for the ongoing activities when they had more knowledge of the activity. According to current theories of event cognition, this is because one’s event model for a new instance of a familiar activity should be informed by both a person’s knowledge structures of the activity, such as their schemas and scripts, and the bottom-up visual information portraying the new instance (Loschky et al., 2020; Zacks et al., 2007). Therefore, we expected that viewers would have a richer event model of the activity when they had more knowledge of the activity, and that these richer event models would guide participants’ attention to the goals of the actor, since successful event comprehension depends upon one’s ability to infer and track an agent’s goals (Zwaan & Radvansky, 1998).

Consistent with our hypothesis, we found that knowledge––both in terms of our familiarity manipulation and self-reported familiarity––with the actions influenced where older adults attended. We also found that young adults did not differ from older adults when they watched the older adult activities; however, they were more likely to attend to goal-relevant information when they performed the activities frequently than when they rarely or never performed the activities. Importantly, we observed no age-related differences in attention to goal-relevant information when older adults have relevant prior knowledge and self-reported familiarity with an everyday activity.

We found differences in viewers’ attention, presumably based on event model differences, whereas other studies have found evidence of the Tyranny of Film. This is likely due to the differences in stimuli between studies. Specifically, our videos lacked many of the filmmaking features that produce strong attentional synchrony, such as cuts, and framing of information in the camera’s view, that are present in Hollywood style films used in prior work (Davis et al., 2021; Hutson et al., 2017; Loschky et al., 2015). The importance of such filmmaking features in producing the Tyranny of Film was suggested by Dorr and colleagues, who found much greater gaze similarity when viewers watched Hollywood movie trailers than “natural videos” (i.e., videos made by setting a video camera on a tripod and filming real world scenes)(Dorr, Martinetz, Gegenfurtner, & Barth, 2010). Thus, our failure to find evidence of the Tyranny of Film suggests that it may depend on the use of standard Hollywood filmmaking techniques.

Importantly, our data are consistent with current theories of event comprehension, which propose that semantic knowledge influences one’s event model (Loschky et al., 2020; Zacks et al., 2007) and hypotheses generated from SPECT, which propose that the viewer’s event model influences their attentional selection (Loschky et al., 2020). We assume that older adults could not use relevant schemas to readily infer the goals of the actor in their event model when they lacked knowledge of the activity, which subsequently impaired their ability to track important goal-relevant information in the videos. Nevertheless, we assume that older adults could utilize their schemas to guide their limited attentional resources to goal-relevant information when the activity was more familiar, which is critical for successful comprehension of everyday events. In addition, our exploratory analysis of self-reported familiarity with the activities suggests that even young adults show an advantage in attention to goal-relevant information. Young adults attended more to the goal-relevant information when they regularly perform the activity than if they rarely or never perform the activity.

It is also possible that we found young adults attended to goal-relevant information, not because it was goal-relevant, but because it was socially relevant. That is, prior work has shown that young people have a propensity to orient their attention to where others are looking (Friesen & Kingstone, 1998; Joyce et al., 2015; Kuhn et al., 2009; Risko et al., 2012). Our AOIs were drawn around objects important for completing the actor’s current goal and, therefore, were often what the actor was looking at in the videos. Conversely, older adults showed a deficit in following the gaze cues of others (Kuhn et al., 2015). This, in turn, could explain why we observed a deficit in older adults’ attention to goal-relevant information in the young adult activities (see Fig. 3). Nevertheless, it does not fully explain why both older and young adults attended similarly to goal-relevant information when they had more self-reported familiarity with an activity. Thus, while a social attention explanation can account for some of our gaze results, it cannot account for the most direct effects of familiarity on gaze.

Older adults may have less precise internal models of actions (Diersch et al., 2012) and this could, in part, explain the age-related deficit in attention to goal-relevant information when the activity was unfamiliar to older adults (see Figs. 3 and 4). Expertise with the actions in the older adult videos may have served as a compensation mechanism in older adults, which produced the lack of an age difference when the activity was familiar to older adults.

It is also possible that older and young adults attend to goal-relevant information because those regions were more visually salient in the film. After all, meaningful areas are also those that tend to be visually salient (Elazary & Itti, 2008; Tatler et al., 2011). However, we do not think visual saliency could explain why young adults attended more to the AOIs than older adults in the young adult activities, but not in the older adult activities. As such, we did not conduct a salience-based analysis using data from this experiment. Future work could assess if older adults attend more to salient regions when they lack relevant knowledge.

We also found that knowledge affected event memory. Like the eye movement results, we found no evidence of an age-related decrement in the older adult’s memory for more familiar older adult activities, but older adults’ memory was significantly worse than young adults for the less familiar young adult activities. That is, age-related deficits arose when older adults lacked relevant knowledge. Consistent with the eye movement results, older adults may have remembered less event information in the young adult activities because they did not have the relevant schemas and scripts for those activities. Prior work has demonstrated that the ability to encode new everyday event information into memory declines with age (Sargent et al., 2013), but one’s knowledge remains intact across the lifespan (Park et al., 2002). Thus, one’s ability to use such knowledge may become increasingly important with age (Umanath & Marsh, 2014) and knowledge may provide an important mechanism for facilitating how information is attended to and later remembered. In the current study, this reliance on prior knowledge seems to have been an advantage for older adults. Prior research has demonstrated a similar null effect sparing of memories for story comprehension (Arbuckle et al., 1990; Miller & Stine-Morrow, 1998; Miller et al., 2004, 2006) and memory for word lists (Castel, 2005). Our study is among the first to demonstrate this effect using more ecologically valid dynamic stimuli (see also Smith et al., 2020 who used slideshows). This suggests that a possible intervention for older adults is to teach them new relevant knowledge that will help them to encode, store, and later retrieve relevant information. We later below discuss such a possible intervention.

Unfortunately, we do not know whether the null effect observed in the older adult activities reflects a boost in memory performance in older adults to the level observed in young adults, or if there is some other possibility for why we observed the null effects. As mentioned previously, some work has suggested that young adults do not rely as much on knowledge as older adults when comprehending texts (Miller & Stine-Morrow, 1998); therefore, young adults may perform well regardless of knowledge. Alternatively, the memory and attention measures we used in the current study may have been insensitive to differences in the older adult activities. We doubt the latter possibility because our memory measures were sensitive to the effects of attention to goal-relevant information (see Figs. 7 and 8) and both young and older adults attended more to goal-relevant information and remembered more when they performed the activity frequently (Daily or Weekly) (e.g., setting up a video game console) compared to when they rarely performed the activity (Monthly or Yearly) or never performed the activity (e.g., balance a checkbook). The latter findings suggest that lacking knowledge produces a deficit in attention to goal-relevant information and memory for both young and older adults. Future research could include more activities to establish whether having knowledge boosts memory in older adults to the level observed in young adults or whether lacking knowledge only hurts memory performance.

In addition, it is also possible that failures in verbal encoding of the actions could explain why older adults showed a memory deficit in the young adult activities, but not in the older adult activities. Specifically, it could account for age-related differences in free recall memory, and to a lesser degree recognition and order memory (see Additional file 1). Observers would not be able to recall actions for activities that they could not verbally describe (e.g., “the thing was plugged into the other thing” vs. “the actor inserted the power supply into the xbox’s power port”). However, this alone could not explain why young adults did not show a deficit in memory for the older adult activities, where they also lacked the relevant terminology to describe the actions (e.g., “the actor compared some numbers to other numbers” vs. “the actor compared the checking balance with the statement”).

Lastly, we found that the degree to which participants attended to goal-relevant information predicted recall memory, but not recognition or order memory. Prior work has demonstrated a strong relationship between eye movements and memory such that people are more likely to successfully remember attended information within a static scene (Hollingworth, 2003; Hollingworth et al., 2001). We found that attention to goal-relevant information predicted the likelihood that the goal-relevant information would successfully be recalled. Note that the AOIs were drawn around the goal-relevant information using the Action Coding System (Schwartz et al., 1991), and we scored the free recall data using the same system. Thus, participants were better able to recall goal-relevant actions to the extent they attended to the goal-relevant actions.

It is also possible that knowledge can affect memory in other ways than by influencing eye movements. Some work has shown that knowledge can influence memory independently from how information is encoded (Newberry et al., 2021; Sargent et al., 2013). This possibility is surprising given all the work that assumes that knowledge affects memory by improving encoding (Anderson & Pichert, 1978; Reingold et al., 2001a, 2001b). For instance, Sargent et al., (2013) found that general measures of event knowledge and event segmentation ability predicted memory independently in a structural equation model after controlling for other possible influences of episodic memory (e.g., age, working memory capacity, etc.). When one has relevant knowledge of a depicted action, they may rely upon knowledge structures to reconstruct the event during recall rather than just relying on what information was attended to or how the event was encoded. This could potentially explain how knowledge affected recognition and order memory in the current experiment (see Additional file 1), but attention to goal-relevant information did not (see Table 4). Such speculations are based upon correlational data and future research should experimentally test this possibility.

A possible limitation with our study is that we used young adult actors in all of the videos. Some work has shown that older adults more accurately infer the emotional state of faces closer in age to themselves (Holland et al., 2019), and they remember own-age faces better than young adult faces (Anastasi & Rhodes, 2005; He et al., 2011). It is possible that older adults would have been more likely to track the goals of the actors if the actors were closer in age to the participants used in the current study. Nevertheless, we do not believe this to be a major concern in the current study since we found that older adults did not differ from young adults in their attention to goal-relevant information or in their event memory when they watched the familiar older adult activities. We should not have found the familiarity advantage for older adults for both attention to goal-relevant information, and memory, if those effects required older adult actors. It is possible however, that the young age of the actors magnified the familiarity effects for the young adults and reduced the familiarity effects for the older adults.

Our results suggest that older participants may rely on their prior knowledge and familiarity to track the goals of people performing everyday activities. These results provide insights for designing interventions that may help improve older adults' ability to attend to new instances of a familiar activity. In addition to training an ability that declines with age (i.e., processing speed, working memory capacity, attentional abilities), future interventions could be developed to teach older adults new activities, which may subsequently influence attention to goal-relevant information and memory in older adults. We are currently exploring this possibility.

## Conclusions

Successful comprehension depends upon the ability to track the goals of others (Zwaan & Radvansky, 1998)). We found that older adults were less likely to track the goals of people performing everyday activities and had poorer memory for the actions when they were less familiar with the activity being performed. But, most importantly, these age-related deficits in attention and memory were not observed when older adults had more familiarity and self-reported experience with the activity. This study demonstrated that knowledge impacts how efficiently people track the goals of others performing everyday activities, and that attention to goal-relevant information could alleviate age-related deficits in event encoding and episodic memory. Thus, older adults, who typically demonstrate a decline in event memory and event understanding (e.g., Sargent et al., 2013; Zacks et al., 2006), may rely on prior knowledge when tracking the goals of other people performing everyday activities.

## Supplementary Information

**Additional file 1.** Supplemental results, tables, and figures.

## Data Availability

All data and materials are available on the Open Science Framework and can be downloaded on the webpage associated with this manuscript: https://osf.io/jkxn4/.

## Footnotes

1 We estimated effect sizes for our linear mixed effects models using formulas provided by Brysbaert and Stevens (2018). Coefficients for each main effect were divided by the square root of the sum of the variances for each random effect (Brysbaert & Stevens, 2018; Westfall, Kenny, & Judd, 2014).

2 This model contains the same predictors of memory performance reported in the Memory section of the manuscript except this model also contains the main effect of the proportion of time spent in the areas of interest as well as the interactions of age group and activity type with the time spent in the AOI.
