# Supplementary material for: Knowledge guides attention to goal-relevant information in older adults
Source: Cogn Res Princ Implic. 2021 Aug 18;6:56. doi: 10.1186/s41235-021-00321-1 (PMC8374018; doi:10.1186/s41235-021-00321-1)
Supplement: Supplementary file 1 — Additional file 1. Supplemental results, tables, and figures. [file 41235_2021_321_MOESM1_ESM.docx]

**Supplemental Materials**

Table 1. Self reports for how often participants perform each of the activities.

|  |  | Setting up a game console | | | | |
| --- | --- | --- | --- | --- | --- | --- |
|  |  | Daily | Weekly | Monthly | Yearly | Never |
| Age Group | Older | 0 | 1 | 0 | 1 | 28 |
|  | Young | 20 | 1 | 5 | 0 | 7 |
|  |  | Setting up a printer | | | | |
|  |  | Daily | Weekly | Monthly | Yearly | Never |
| Age Group | Older | 0 | 1 | 3 | 18 | 8 |
|  | Young | 0 | 1 | 1 | 25 | 4 |
|  |  | Balancing a Checkbook | | | | |
|  |  | Daily | Weekly | Monthly | Yearly | Never |
| Age Group | Older | 2 | 10 | 11 | 2 | 5 |
|  | Young | 0 | 0 | 9 | 9 | 15 |
|  |  | Planting Flowers | | | | |
|  |  | Daily | Weekly | Monthly | Yearly | Never |
| Age Group | Older | 5 | 13 | 6 | 6 | 0 |
|  | Young | 0 | 2 | 5 | 19 | 7 |

**Psychometric Tasks.** After viewing each video, participants completed various psychometric measures. The psychometric tests included tests of processing speed, script knowledge, vocabulary, and working memory. The order of the psychometric tests was not counterbalanced. Each test lasted approximately 5 minutes.

*Script generation*. To assess event knowledge, participants were given 2 minutes and 30 seconds to write down in order, all the steps involved in each of 2 everyday activities (Going to Dinner and Scuba Diving) (Rosen, Caplan, Sheesley, Rodriguez, & Grafman, 2003). We scored script generation performance by counting the number of normative steps using the scripts provided by Rosen et al., (2013) that each participant correctly mentioned in descriptions of how to perform each activity.

*Vocabulary*. Participants were given 4 minutes to complete a computerized version of the Shipley Vocabulary Test (Zachary & Shipley, 1986). On each trial, an underlined target word appeared at the top of the screen. Five multiple choice answers appeared below the target. Participants were asked to click the letter of the answer that had the closest meaning to the target word. Performance was scored as the number correct within 5 minutes.

*Processing Speed*. Three measures of processing speed were administered to the participants. In the pattern comparison task, participants were asked to indicate with a button press whether pairs of patterns presented on the computer screen were the same or different (Earles & Salthouse, 1995). The letter comparison task was similar to the pattern comparison task. Pairs of letter string were presented on the computer screen. Participants had 60 seconds to indicate with a button press whether the 2 strings of letters were the same or different. Letter strings consisted of randomly selected consonants and ranged in length from 3 characters to 9 characters. The last measure of processing speed we administered to participants was the Digit Symbol Substitution test (Wechsler, 1955). The task consists of nine digit-symbol pairs. Under each digit, participants’ task was to draw the symbol that corresponded to the digit from a table of digits and symbols. Performance for each processing speed task was scored as the number of correctly completed trials in 60 seconds.

*Working Memory*. Participants completed a shortened version of the rotation span task (Foster et al., 2015). The Rotation Span task is very similar to other measures of working memory capacity. Participants were shown a rotated letter in the center of the screen. The letter was either presented correctly or it was a mirror image. Participants were asked to judge whether the letter was mirror reversed or normal after mentally rotating it to be upright. The to-be-remembered items for this test were arrows of either short or long length. Arrows pointed to one of eight different locations on the screen. Participants were asked to remember the length and location of the arrows in the correct order. The rotation-arrow sequence varied from three to five sets per trial. Participants completed two blocks of the rotation span task. Scores were calculated by calculating the partial score for each participant. This was done by summing the number of arrows each participant correctly recalled in the correct order (Turner & Engle, 1989). We also calculated the absolute score for each participant by counting the number of to-be-remembered arrows only from completely correctly produced sets.

*Segmentation Agreement*. Participants watched each of the videos in the same order of the original viewing, but this time they performed the segmentation task, and their eyes were not tracked. For the segmentation task, participants were instructed to press the spacebar on a keyboard whenever they felt that “one meaningful unit of activity ends, and another begins” (Newtson, 1973; Zacks, Tversky, & Iyer, 2001). They began by segmenting the practice video. If they identified fewer than 3 boundaries (this value was not known to participants), then they were told that participants typically identify more units than what they did. They then were told they would have to complete the task again by re-watching the video. Participants segmented the remaining experimental videos after successful segmentation of the practice video.

To calculate segmentation agreement, we recorded the frame number when a participant identified an event boundary. The probability of two participants segmenting at the exact same frame is very low even if they both perceive the same boundary. Thus, we fit a 1 second (Kurby & Zacks, 2011) Gaussian Kernel function to each participant’s button presses for each video, resulting in a probability ranging from 0 to 1 for each frame that the frame was a boundary. The main advantage of our agreement calculation method over other methods is that it allowed us to treat event boundary perception as probabilistic rather than categorical. Next, we averaged the event boundary probabilities of each frame across the participants within the familiar group (older adults in the older adult videos and young adults in the young adult videos) to get the *normative boundaries*. Finally, we correlated each individual’s segmentation probabilities with the normative boundaries. A leave-one-out procedure was used to calculate agreement for members of the familiar group so that each participant’s own probabilities were not included in the normative distribution. Each participant received one correlation value for each of the 4 videos.

**Percent of fixations in areas of interest**. To examine if knowledge influenced attention to goal relevant information, we fit a linear mixed model to the proportion of fixations participants had in the areas of interest. To calculate this proportion, we took the ratio between the total number of fixations in the areas of interest for each participant and divided it by the total number of fixations for that subject. This was done to account for differences in the number of fixations acquired from each participant. The model included the average size of the AOIs, standardized at its mean, the age group of the participants (effect coded as Young = -1 and Older = +1), the type of activity (effect coded as Young Activity = -1 and Older Activity = +1), and the interaction between the age group and activity as fixed effects. We determined the random effect structure of the model by comparing models with the same fixed, but different random effect structures. The first model was the “maximal model”, and then reduced it until we found that it was a statistically better fit of the data than a reduced model. All of the models contained the participant intercepts and the videos as random effects. We allowed the effect of age group to vary as a random slope effect with video, and we allowed the effect of activity type to vary as a random slope effect with the participant intercept in the first model. Unfortunately, the maximal model failed to converge. In the second model, we removed the effect of age group as a random effect, so that the second model only contained the by-participant slope effect, the by-participant intercept, and the video intercept as random effects. We removed the by-participant slope of activity type in the third model, so that it only contained the participant and video intercepts as random effects. The second and third models did not significantly differ, *χ*^2^(2) = 0.89, *p* = .64; therefore, we retained the third model.

We found an age-related difference in the young adult, but not the older adult activities. Participants made more fixations in the AOIs when they were larger than when they were smaller, *β* = 0.10, *SE* = 0.007, *t* = 14.45, *p* < .001. We also found a significant effect of age group such that young adults (*M* = 0.42, *SE* = 0.01) made more fixations in the goal-relevant areas of interest than older adults (*M* = 0.38, *SE* = 0.01), *β* = -0.02, *SE* = 0.008, *t* = -2.53, *p* = .01, *d* = 0.25. We also found a significant effect for activity type [Young Adult Videos (*M* = 0.36, *SE* = 0.01); Older Adults Videos (*M* = 0.44, *SE* = 0.01)], *β* = 0.04, *SE* = 0.007, *t* = 6.17, *p* <.0001, *d* = 0.51. As evident in Figure 1, we also observed a significant interaction between age group and activity type, *β* = 0.02, *SE* = 0.004, *t* = 3.79, *p* < .001, *d* = 0.19. Older and young adults did not significantly differ in their attention to goal relevant information for the older adult videos [Young Adults (*M* = 0.45, *SE* = 0.01); Older Adults (*M* = 0.44, *SE* = 0.01)], *β* = -.008, *SE* = .02, *t* = -0.47, *p* = .99; however, young adults attended more to goal relevant information than older adults in the young adult videos [Young Adults (*M* = 0.40, *SE* = 0.01); Older Adults (*M* = 0.32, *SE* = 0.01)], *β* = -0.07, *SE* = .02, *t* = -4.00, *p* = .0003. Thus, older adults did not differ in their attention to goal-relevant information when they could rely upon prior knowledge to inform their understanding of the video, but they attended less to goal-relevant information when they could not rely on prior knowledge.


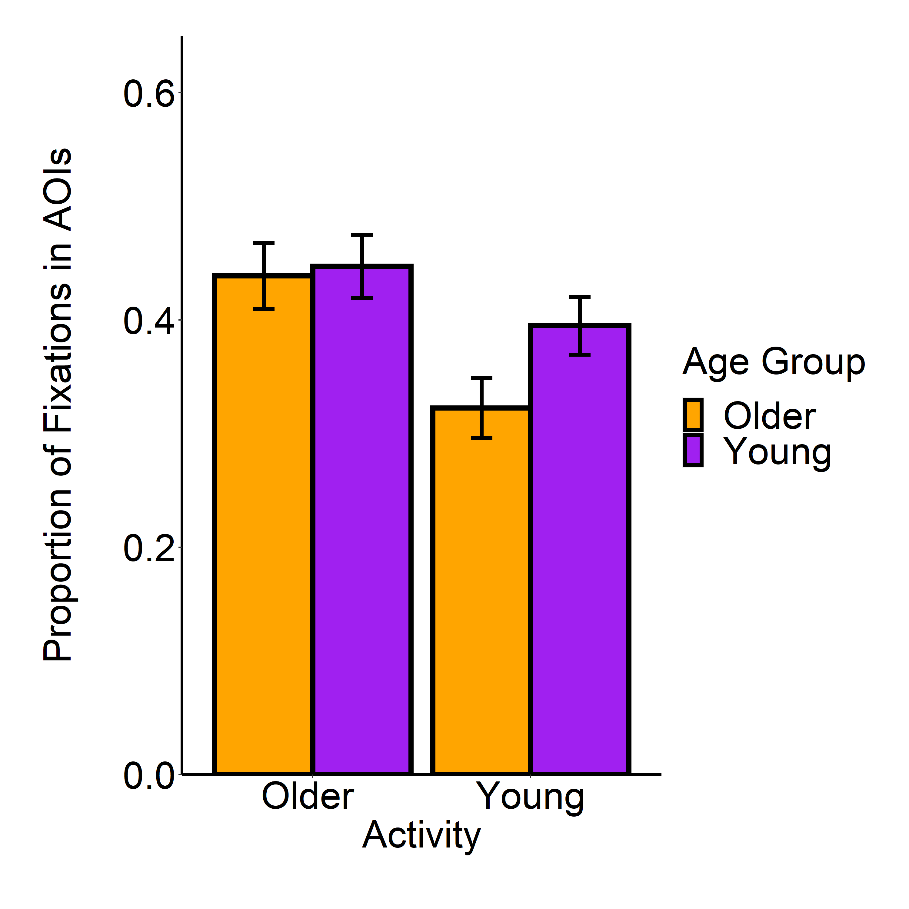


*Figure 1*. Proportion of fixations in goal relevant areas of interest as a function of age group and activity type. Error bars represent 1 standard error around the estimated means.

**Memory Measures**. Participants completed three different memory tests after watching each video. We measured participants’ free recall, recognition, and order memory. Performance on each measure was moderately to strongly correlated (range of *r* = 0.26 to 0.81).

*Free Recall Memory*. Event recall was scored as the number of correctly recalled actions. We conducted two different analyses. We first assessed the effect of knowledge on the number of correctly recalled A1 actions and then we assessed the effect of knowledge and age on the number of correctly recalled A2 actions. We ran a mixed effects Poisson regression because we repeatedly measured from each participant and the dependent measure was count data. Both models contained Age group, Activity type, and their interaction as fixed effects. Participant and video were both treated as random effects as was done in the primary manuscript.

We found no age-related differences in the older adult familiar activities, but young adults recalled more than older adults in the young adult activities. Overall, young adults (*M* = 13.85, *SE* = 1.56) recalled significantly more A1 actions than older adults (*M* = 8.77, *SE* = 1.02), *β* = -0.23, *SE* = 0.05, *z* = -4.25, *p* <.001. We did not observe as significant effect of activity type [Young Adults Activities (*M* = 10.20, *SE* = .1.38); Older Adults Activities (*M* = 11.90, *SE* = .1.59)], *β* = 0.07, *SE* = 0.09, *z* = 0.85, *p* = .39. We found a significant two-way interaction between age group and activity type, *β* = 0.11, *SE* = 0.02, *z* = 6.00, *p* < .001. Young adults (*M* = 14.35, *SE* = 2.06) recalled significantly more in the young adult activities than older adults (*M* = 7.29, *SE* = 1.09), *β* = 0.68, *SE* = 0.12, *z* = 5.92, *p* < .001, however both age groups recalled a similar number of actions in the older adult videos [Young Adults (*M* = 13.36, *SE* = 1.92); Older Adults (*M* = 10.56, *SE* = 1.55)], *β* = -0.24, *SE* = 0.11, *z* = -2.09, *p* = .07.

Results were similar for recall of A2 units. Young adults recalled significantly more actions in the young adult videos, but we did not find an age-related deficit for the older adult activities. Overall, we found a significant effect for the age group [Young Adults (*M* = 12.19, *SE* = 0.57); Older Adults (*M* = 8.83, *SE* = 0.45)], *B*= -0.16, *SE* = 0.03, *z* = -4.69, *p* <.001. Participants recalled significantly more A2 actions from the older adult videos [Young Adult Videos (*M* = 9.79, *SE* = 0.40); Older Adults Videos (*M* = 10.99, *SE* = 0.43)], *β* = 0.06, *SE* = 0.02, *z* = 2.94, *p* = .003. Consistent recall of the A1 actions, we observed a significant interaction between age group and activity, *β*= 0.12, *SE* = 0.02, *z* = 6.20, *p* <.001; whereby, young adults recalled significantly more actions in the young adult videos [Young Adults (*M* = 13.00, *SE* = 0.67); Older Adults (*M* = 7.38, *SE* = 0.46)], *β*= 0.57, *SE* = 0.08, *z* = 7.00, *p* < .001, but not for the older adult activities [Young Adults (*M* = 11.44, *SE* = 0.61); Older Adults (*M* = 10.55, *SE* = 0.60)], *β* = -0.08, *SE* = 0.08, *z* = -1.04, *p* = .59. Thus, older adults recalled a similar number of actions as young adults when they could rely on prior knowledge.

*Recognition Memory*. Recognition memory was recorded as a 1 if the participant got the trial correct and a 0 if the participant got the trial incorrect. We used a logistic mixed effects regression to analyze the effect of knowledge on recognition memory using the trial-by-trial data. Age group, activity type, and their interaction were treated as fixed effects. Participant and video were both treated as random effects.

Results for recognition memory were comparable to that of free recall memory. Young adults had better memory than older adults for the young adult activities, but the age groups did not differ in their recognition memory for the older adult activities. Overall, we found a main effect for age group [Young Adults (*M* = 0.70, *SE* = 0.05); Older Adults (*M* = 0.64, *SE* = 0.06)], *β* = -0.14, *SE* = 0.05, *z* = -2.57, *p* = .01, but not for activity type, [Young Adult Activities (*M* = 0.64, *SE* = 0.08); Older Adult Activities (*M* = 0.70, *SE* = 0.07)], *β* = 0.14, *SE* = 0.23, *z* = 0.62, *p* = .54. We also found a marginally significant interaction between age and activity type, *β* = 0.06, *SE* = 0.03, *z* = 1.91, *p* = .05.

We probed this marginally significant interaction and found that young adults had better recognition memory for the young adult activities [Young Adults (*M* = 0.68, *SE* = 0.07); Older Adults (*M* = 0.59, *SE* = 0.08)], *β* = 0.39, *SE* = 0.12, *z* = 3.24, *p* = .002; however, the age groups did not differ in their recognition memory of the older adult activities, [Young Adults (*M* = 0.72, *SE* = 0.07); Older Adults (*M* = 0.69, *SE* = 0.07)], *β* = 0.16, *SE* = 0.12, *z* = -1.24, *p* = .43.

*Order Memory*. Order memory was scored the same as recognition memory. We again used a logistic mixed effects regression to analyze the effect of knowledge on order memory. The fixed and random effects were the same as in the model for recognition memory.

Results for order memory were very similar to the results we observed for free recall and recognition memory. Young adults had better memory than older adults in both activities; however, the difference in their order memory was larger in the young adult videos. Overall, young adults (*M* = 0.91, *SE* = 0.26) had significantly better order memory than older adults (*M* = 0.85, *SE* = 0.04), *β* = -0.25, *SE* = 0.05, *z* = -4.62, *p* <.001. We did not observe a significant difference between activities [Young Adult Activities (*M* = 0.88, *SE* = 0.05); Older Adult Activities (*M* = 0.89, *SE* = 0.04)], *β* = 0.06, *SE* = 0.30, *z* = 0.19, *p* = .85; however, we did observe a significant interaction between age group and activity type, *β* = 0.11, *SE* = 0.02, *z* = 4.88, *p* < .001. Young adults had significantly better order memory for both activities; however, the difference between the age groups was smaller for the older adult activities [Young Adults (*M* = 0.90, *SE* = 0.04); Older Adults (*M* = 0.87, *SE* = 0.05)], *β* = -0.28, *SE* = 0.12, *z* = -2.34, *p* = .04 than the young adult activities [Young Adults (*M* = 0.91, *SE* = 0.03); Older Adults (*M* = 0.83, *SE* = 0.06)], *β* = -0.73, *SE* = 0.12, *z* = -6.15, *p* < .001.

References

Earles, J. L., & Salthouse, T. A. (1995). Interrelations of age, health, and speed. *The Journals of Gerontology Series B: Psychological Sciences and Social Sciences, 50*(1), P33-P41.

Foster, J. L., Shipstead, Z., Harrison, T. L., Hicks, K. L., Redick, T. S., & Engle, R. W. (2015). Shortened complex span tasks can reliably measure working memory capacity. *Memory & Cognition, 43*(2), 226-236.

Kurby, C. A., & Zacks, J. M. (2011). Age differences in the perception of hierarchical structure in events. *Memory and Cognition, 39*, 75-91.

Newtson, D. (1973). Attribution and the unit of perception of ongoing behavior. *Journal Of Personality And Social Psychology, 28*(1), 28-38.

Rosen, V., Caplan, L., Sheesley, L., Rodriguez, R., & Grafman, J. (2003). An examination of daily activities and their scripts across the adult lifespan. *Behavior Research Methods, Instruments, & Computers, 35*(1), 32-48.

Turner, M. L., & Engle, R. W. (1989). Is working memory capacity task dependent? *Journal of Memory and Language, 28*(2), 127-154.

Wechsler, D. (1955). *Wechsler adult intelligence scale*: Psychological Corporation New York.

Zachary, R. A., & Shipley, W. C. (1986). *Shipley institute of living scale: Revised manual*: WPS, Western Psychological Services.

Zacks, J., Tversky, B., & Iyer, G. (2001). Perceiving, remembering, and communicating structure in events. *Journal of Experimental Psychology-General, 130*(1), 29-58.
